# Targeting the E2F1/Rb/HDAC1 axis with the small molecule HR488B effectively inhibits colorectal cancer growth

**DOI:** 10.1038/s41419-023-06205-0

**Published:** 2023-12-07

**Authors:** Namin Duan, Xiaohui Hu, Huiran Qiu, Rui Zhou, Yuru Li, Wenxia Lu, Yamin Zhu, Shuang Shen, Wenhui Wu, Feifei Yang, Ning Liu

**Affiliations:** 1 Department of Chemistry, College of Food Science and Technology, Shanghai Ocean University, Shanghai, China; 2 School of Biological Science and Technology, University of Jinan, Jinan, P.R. China; 3 Marine Biomedical Science and Technology Innovation Platform of Lingang Special Area, Shanghai, China; 4 National Experimental Teaching Demonstration Center for Food Science and Engineering, Shanghai Ocean University, Shanghai, China; 5 Shanghai Jiao Tong University Affiliated Sixth People’s Hospital, Shanghai, China; 6 Department of Marine Bio-Pharmacology, College of Food Science and Technology, Shanghai Ocean University, Shanghai, China; 7 Jerome Lipper Multiple Myeloma Center, Department of Medical Oncology, Dana-Farber Cancer Institute, Harvard Medical School, Boston, USA

**Keywords:** Cancer, Cancer therapy, Drug development

## Abstract

Colorectal cancer (CRC), the third most common cancer worldwide, remains highly lethal as the disease only becomes symptomatic at an advanced stage. Growing evidence suggests that histone deacetylases (HDACs), a group of epigenetic enzymes overexpressed in precancerous lesions of CRC, may represent promising molecular targets for CRC treatment. Histone deacetylase inhibitors (HDACis) have gradually become powerful anti-cancer agents targeting epigenetic modulation and have been widely used in the clinical treatment of hematologic malignancies, while only few studies on the benefit of HDACis in the treatment of CRC. In the present study, we designed a series of small-molecule Thiazole-based HDACis, among which HR488B bound to HDAC1 with a high affinity and exerted effective anti-CRC activity both in vitro and in vivo. Moreover, we revealed that HR488B specifically suppressed the growth of CRC cells by inducing cell cycle G0/G1 arrest and apoptosis via causing mitochondrial dysfunction, reactive oxygen species (ROS) generation, and DNA damage accumulation. Importantly, we noticed that HR488B significantly decreased the expression of the E2F transcription factor 1 (E2F1), which was crucial for the inhibitory effect of HR488B on CRC. Mechanistically, HR488B obviously decreased the phosphorylation level of the retinoblastoma protein (Rb), and subsequently prevented the release of E2F1 from the E2F1/Rb/HDAC1 complex, which ultimately suppressed the growth of CRC cells. Overall, our study suggests that HR488B, a novel and efficient HDAC1 inhibitor, may be a potential candidate for CRC therapy in the future. Furthermore, targeting the E2F1/Rb/HDAC1 axis with HR488B provides a promising therapeutic avenue for CRC.

## Introduction

Colorectal cancer (CRC) is one of the leading causes of cancer lethality worldwide with more than 1.6 million deaths projected in 2040 [1]. At present, the treatment of CRC mainly includes surgery, radiation therapy, and chemotherapy, however, treatment-related side effects and complications impact the postoperative quality of life. Patients after surgery are at risk of bowel dysfunction [2]. In addition, the chemotherapy agents are poor selectivity and have a killing effect on normal cells while killing tumor cells [3]. Moreover, drug resistance to first-line treatment drugs is also a crucial hurdle to curing this malignancy [4]. Consequently, there is an urgent need for developing more effective and better-tolerated anti-CRC therapeutic agents to improve the therapeutic efficacy of CRC treatment and patient outcomes.

Epigenetic modifications include DNA methylation, post-translational modifications of histone, and non-coding RNAs, while the failure of the normal epigenetic modifications in epigenetic processes can cause altered gene function and malignant cellular transformation [5]. As an important epigenetic eraser, histone deacetylases (HDACs) remove acetyl groups from the N-acetylated lysine residues in the tail of histones, resulting in chromatin condensation and impacting gene expression [6]. Besides histones, numerous nonhistone proteins that are essential for cell cycling, apoptosis pathways, DNA repair, transport, and transcriptional regulation are all regulated by acetylation/deacetylation [7]. In addition, the abnormal overexpression of HDACs is also strongly correlated with the occurrence and progression of CRC and several other cancers [8]. To date, HDAC inhibitors (HDACis) such as SAHA, FK228, belinostat, panobinostat, and chidamid approved have been approved by the U.S. Food and Drug Administration and demonstrated favorable anti-cancer activities against hematological malignancy. However, toxic side effects and readily produce drug resistance limit HDACis application in cancer therapy [9]. Moreover, numerous HDACis exhibit weak clinical efficiency in treating solid tumors due to poor isoform selectivity, high toxic responses, narrow therapeutic indices, and a deficiency of reliable biomarkers [10]. Hence, it is necessary to develop novel HDACis with improved therapeutic effects and decreased toxicities. Here, we designed and synthesized a novel series of Thiazole-based HDACis and assessed their biological activities in multiple kinds of cancer cells. The obtained candidate compound HR488B showed a high anti-proliferative effect against CRC cells. Meanwhile, the regulatory mechanisms underlying HDACis action and the potential therapeutic targets for CRC therapy are also worthy of further exploration.

E2F transcription factor 1 (E2F1) belongs to the E2F family of transcription factors, which plays key roles in regulating cell cycle, differentiation, and apoptosis in a variety of human cancers due to its abnormal overexpression as well as its interactions with effect factors including Bax, Cyclin D1, CDK4, PUMA, and so on [11, 12]. In recent years, numerous studies have found that pharmacological and genetic inhibition of E2F1 significantly inhibited tumor growth in CRC and E2F1 has also been revealed to participate in the metastasis and chemoresistance of CRC [13–15]. Mechanistically, the E2F1/Rb pathway is likely critical for the regulation of cell growth and the development of cancer. Previous studies have displayed that the low phosphorylation of Rb inhibited the release of E2F1 from Rb, thus hindering the transcription activity of E2F1 [16]. Moreover, class I HDACs (HDAC1-3) cooperate with Rb family members and E2Fs in the regulation of genes involved in cell cycle progression [17]. It has been proposed that these enzymes are recruited to E2F1 target promoters where they deacetylate histones and modify chromatin structure [18]. An analysis showed that E2F1 partly localized to mitochondria, where it promoted mitochondrial outer membrane permeabilization, while mitochondria controlled various cellular parameters including energy production, oxidation-reduction balance, and reactive oxygen species (ROS) production, contributing to the initiation of apoptosis [19, 20]. Therefore, the identification of novel small molecules targeting the E2F1/Rb/HDAC1 complex may provide a more effective therapeutic strategy for cancer.

Herein, we identified a novel HDAC1 inhibitor HR488B that obviously suppressed CRC development and progression both in vitro and in vivo. Further mechanistic studies revealed that HR488B regulated the expression of E2F1 protein by repressing Rb phosphorylation, alleviating the E2F1/Rb/HDAC1 complex disassembly, inducing cell cycle arrest, DNA damage, and cell apoptosis, and finally resulting in CRC inhibition. Collectively, our results suggested that HR488B might be a promising candidate for CRC treatment and that targeting the E2F1/Rb/HDAC1 complex provides a rationale for future evaluation of HR488B as a potential drug in CRC.

## Results

### Identification of HR488B as a novel HDACi

**Fig. 1:**
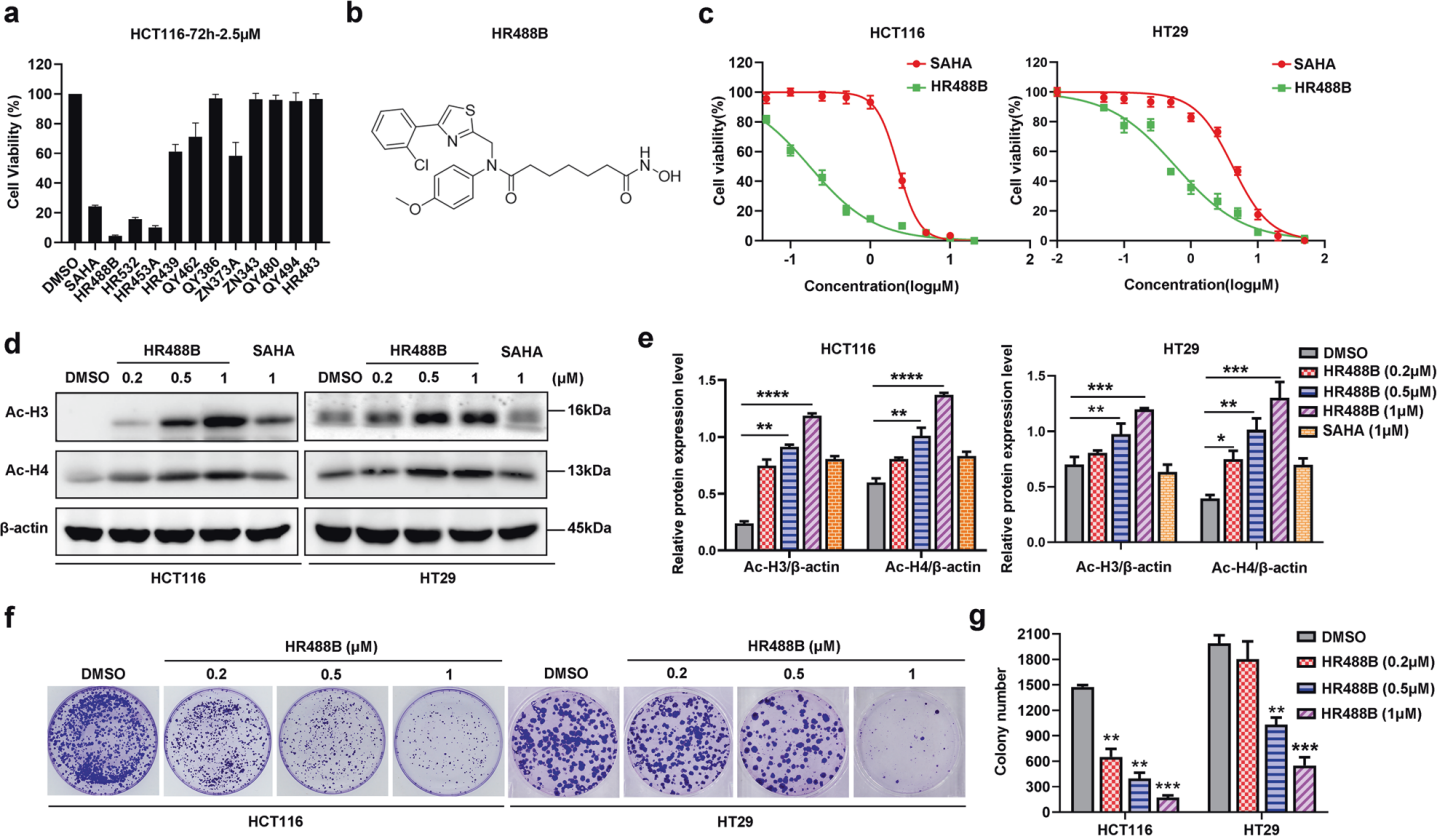
Identification of HR488B. **a** HCT116 cells were treated with the indicated compounds (2.5 μM) for 72 h. Cell viability was obtained from the CCK-8 assay. SAHA was used as a positive control. **b** Chemical structure of the HDACi HR488B. **c** HCT116 and HT29 were treated with the indicated concentrations of HR488B (0.01, 0.05, 0.1, 0.25, 0.5, 1, 2.5, 5, 10, and 20 μM) or DMSO for 72 h, and cell viability was assessed using CCK-8 assay and shown as relative viability compared to the untreated control. Each test was performed in triplicate. **d** HCT116 and HT29 cells were treated with DMSO, SAHA (1 μM), or different doses of HR488B (0.2, 0.5, and 1 μM) for 24 h. The expression of acetylated H3/H4 protein was determined by Western blot analysis, and β-actin was detected as the endogenous loading control, accordingly. **e** The statistical result of (**d**). **f** Colony-forming abilities of HCT116 and HT29 cells after treating cells with the indicated concentrations of DMSO or HR488B (0.2, 0.5, and 1 μM) for 14 days. **g** The statistical result of (**f**). All data are shown as mean ± SD, *n* = 3, two-way ANOVA, *p < 0.05, **p < 0.01, ***p < 0.001, ****p < 0.0001.

HDACis have been proven to be effective anti-tumor agents at various stages of the investigation [21]. However, the approved HDACis display a lack of visible efficacy against solid tumors despite the promising results in the treatment of cutaneous T cell lymphoma and peripheral T cell lymphoma [22]. To specifically identify new potential agents for CRC, we screened an *in-house* library of small-molecule hydroxamate-based HDACis against CRC cells. SAHA, the first hydroxamic acid HDACi, was selected as a positive control in our study. We firstly investigated the inhibitory activity of HDACis in the *in-house* library using cell viability assay. As shown in Fig. 1a, HR488B displayed the optimum inhibitory effect on the cell viability of HCT116 cells, suggesting a special inhibitory action of HR488B in CRC cells. The chemical structure of HR488B was shown in Fig. 1b, and the synthesis scheme and the characterization data of HR488B were shown in Figs. S1, S2. To further explore the characteristics of HR488B on CRC, the IC_50_ values of HR488B on different CRC cell lines, HCT116 and HT29, were determined. Figure 1c showed that HR488B exhibited a substantially stronger inhibitory effect against HCT116 (IC_50_ = 0.17 μM) and HT29 (IC_50_ = 0.59 μM) than SAHA (IC_50_ = 2.13 μM). Meanwhile, to confirm the role of HR488B in cancer cell growth, the cytotoxic effect of HR488B on other cancer cell lines (A549, H1299, HepG2, MCF-7) and normal renal epithelial 293T cells were also examined. We found that HR488B had a much stronger inhibitory effect on HCT116 cells than other cancer cells (Fig. S3a–e), suggesting a special inhibitory action of HR488B in CRC cells. Importantly, we did not observe any significant cellular toxicity of 293T cells post receiving a low concentration of HR488B treatment (Fig. S3f), which demonstrated the low toxicity of HR488B. In addition, the results of analysis of both cell lines revealed that HR488B significantly increased the acetylation of H3 and H4 in a dose-dependent manner compared with SAHA (Fig. 1d, e). Finally, we examined the effect of HR488B on cell colony formation. The results demonstrated that HR488B obviously reduced the number and size of the colonies of HCT116 cells and HT29 cells in a dose-dependent manner, which confirmed its remarkable inhibitory effects on CRC cells (Fig. 1f, g). These data suggest that HR488B is a potent HDACi effectively inhibiting the proliferation of CRC cells, and we decided to focus on HR488B in the following confirmatory experiments.

### HR488B potentially and selectively inhibits HDAC1

**Fig. 2:**
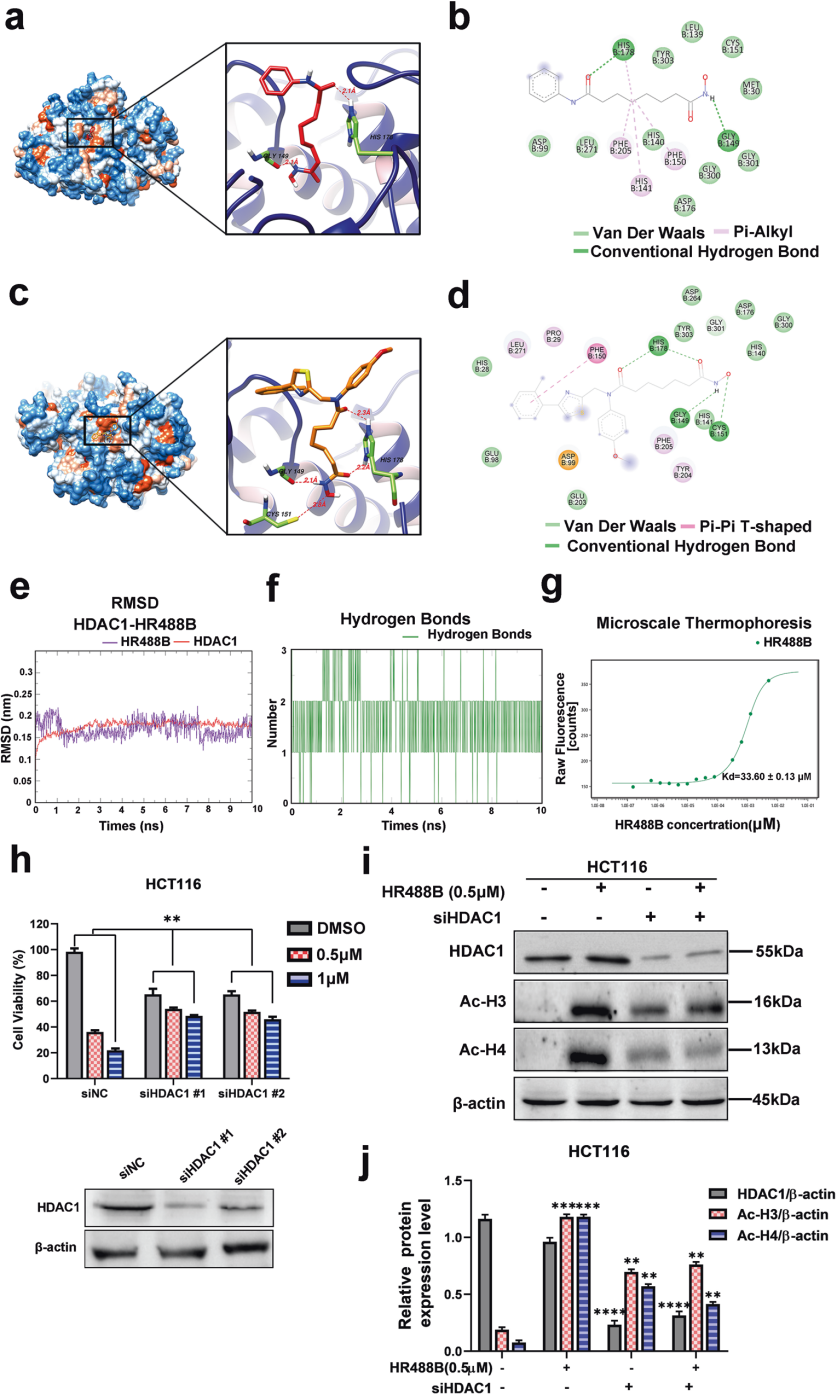
HDAC1 inhibition and selectivity by HR488B. **a** The 3D binding mode of SAHA (red) in the active site of HDAC1. The protein and ligand SAHA and HR488B are shown by cartoon and stick, respectively, with key residues labeled and demonstrated as green sticks, and the hydrogen bonds are labeled by red dashed lines. **b** Diagrammatic illustration of interaction between HDAC1 binding site residues and SAHA by BIOVIA Discovery Studio Visualizer software. Ligand is presented by gray line, green dashed line is conventional hydrogen bonds, light green dashed line is Van der Waals, and light pink dashed line is Pi-Alkyl. **c** The 3D binding mode of HR488B (orange) in the active site of HDAC1. The protein and ligand SAHA and HR488B are shown by cartoon and stick, respectively, with key residues labeled and demonstrated as green sticks, and the hydrogen bonds are labeled by red dashed lines. **d** 2D binding mode of HR488B into the HDAC1. Ligand is presented by gray line, green dashed line is conventional hydrogen bonds, light green dashed line is Van der Waals, and pink dashed line is Pi-Pi T-shaped. **e** Root mean square deviation (RMSD) values of HDAC1 (red) and in complex with HR488B (purple) over 10 ns. **f** The number of hydrogen bonds formed between the HR488B and HDAC1 during the structural rearrangement. **g** Microscale thermophoresis (MST) analysis of the binding affinity between HR488B and HDAC1. The measured Kd value is shown. **h** HCT116 cells were transfected with siRNAs targeting HDAC1 (siHDAC1#1 and siHDAC1#2) or control siRNA (siNC), and 48 h later, cells were treated with or without HR488B (0.5 and 0.1 μM) for 24 h. Cell viability was assessed with the CCK-8 assay. **i** HCT116 cells were transfected with siRNAs targeting siHDAC1 or siNC, and 48 h later, cells were treated with DMSO or HR488B (0.5 μM) for 24 h, and the expression of HDAC1, Ac-H3, and Ac-H4 protein was determined by Western blot analysis, and β-actin was detected as the endogenous loading control, accordingly. **j** The statistical result of (**i**). All data are shown as mean ± SD, *n* = 3, two-way ANOVA, *p < 0.05, **p < 0.01, ***p < 0.001, ****p < 0.0001.

HR488B was designed as a hydroxamate-based bis-substituted aromatic amide HDACi in Fig. S1. To evaluate the potency and subtype selectivity of HR488B, we conducted in vitro assays against several subtypes of HDAC enzymes, including HDACs enzymes of class I (HDAC1, 2, and 8), and class IIb (HDAC6). The results presented in Table 1 demonstrated that HR488B exhibited good selectivity against HDAC1 over HDAC2 (IC_50_ = 1.24 nM against HDAC1, IC_50_ = 10.42 nM against HDAC2), and showed low activity against HDAC8 (IC_50_ > 10 μM) and HDAC6 (IC_50_ > 10 μM). Notably, HR488B demonstrated significantly higher potency against HDAC1 than SAHA, with nearly 12-fold higher potency (IC_50_ = 1.24 nM for HR488B vs. IC_50_ = 15.12 nM for SAHA). To better elucidate the effective HDAC inhibitory activity of HR488B, molecular docking was performed to explore the binding mode of HR488B with HDAC1 (PDB ID:4BKX). As illustrated in Fig. 2a, c, HR488B and SAHA shared similar combination patterns with HDAC1, while the hydroxamic acid group connected to the aromatic amide of HR488B could more stably enter the hydrophobic pocket of the surface region of HDAC1. Given the extensive structural and functional similarities between HDAC1 and HDAC2, we further performed molecular docking of the HDAC2(PDB ID: 7ZZS) to HR488B. The result showed that the aromatic amide of HR488B did not enter the active pocket of HDAC2 (Fig. S4a). Moreover, a π–π stacking was observed between the phenyl of HR488B and PHE150 sitting in the surface region of HDAC1 contributing to the enhanced stability of the binding model between HR488B and HDAC1 (Fig. 2d). Compared to SAHA, four hydrogen bond interactions were formed between hydroxamic acid group of HR488B and residues surrounding the active site, including HIS178, GLY149, and CYS151, which were located around the Zinc binding group and contributed to the stronger binding capacity of HR488B and HDAC1 (Fig. 2b, d). On the other hand, the hydroxamic acid group of HR488B was unable to access the active site of HDAC2, and no analogous hydrogen bonding interactions were formed, indicating its weaker binding capacity towards HDAC2 (Fig. S4b). In addition, compared to SAHA’s binding affinity with HDAC1 (a docking score of −4.82) and HR488B’s binding affinity with HDAC2 (a docking score of −4.98), HR488B exhibited stronger binding affinity to HDAC1 with a docking score of −6.73. This further demonstrated the superiority of HR488B combined with HDAC1 posture (Figs. S4a, S5a). To further validate the stability of the binding pose mentioned above, molecular dynamics simulations of HDAC1-HR488B complex in aqueous solution were conducted using GROMACS (2020.6) with Amber99 force field [23]. With 10 ns simulation, root mean square deviation (RMSD) values of HDAC1 and HR488B were calculated, respectively, and long-term simulation demonstrated that the binding mode of HDAC1-HR488B was stable since 3 ns (Fig. 2e, f). Compared with molecular docking results, the amount of stable hydrogen bonds between HDAC1 and HR488B was two, which was contributed to TYR303, HIS140 residues and occupied 71% and 57.4%, respectively, during the whole process of simulation (Fig. S5b). Moreover, HR488B formed two carbon hydrogen bonds with HIS141 and GLY149 residues at 3 ns, 5.5 ns, and 8 ns which played an essential role in the biological function of histone deacetylase [24] (Fig. S5c–e). To validate the interaction between HR488B and HDAC1, we analyzed their binding affinity using MST and fluorescence-labeled HDAC1. The obtained dissociation constant (Kd) of 33.6 ± 0.13 μM from the affinity curve suggested that HR488B stably bound to HDAC1 with a strong affinity (Fig. 2g). These above results indicated that HR488B had a high affinity close to the active site pocket of HDAC1 and inhibited the catalytic activity of HDAC1.

**Table 1:** In vitro inhibition of HDACs isoforms of HR488B.

Compound	IC_50_(nM)			
	Class I			Class IIb
	1	2	8	6
HR488B	1.24 ± 0.05	10.42 ± 0.07	>10,000	>10,000
SAHA	15.12 ± 0.46	19.21 ± 0.05	>10,000	>10,000

IC_50_: Half maximal inhibitory concentration.

To further determine the target specificity of HR488B, we knocked down HDAC1 to confirm whether the inhibitory effect of HR488B on HCT116 cells is affected. In the siNC group, treatment with HR488B led to a remarkable downregulation of HCT116 cell viability compared to the DMSO-treated group. In siHDAC1 cells, although cell proliferation was still inhibited to a certain extent, no significant change in cell viability was observed between the HR488B-treated and DMSO-treated groups (Fig. 2h). Correspondingly, compared to the DMSO-treated siHDAC1 cells, we observed no obvious change in the protein expression levels of Ac-H3 and Ac-H4 observed in HR488B-treated siHDAC1 cells (Fig. 2i, j). The collective results suggested that the inhibitory effect of HR488B on HCT116 cells was significantly reduced upon HDAC1 knockdown, confirming that HR488B exerts its anti-tumor activity via inhibition of HDAC1.

### HR488B significantly suppresses the tumor growth in an HCT116 xenograft model

**Fig. 3:**
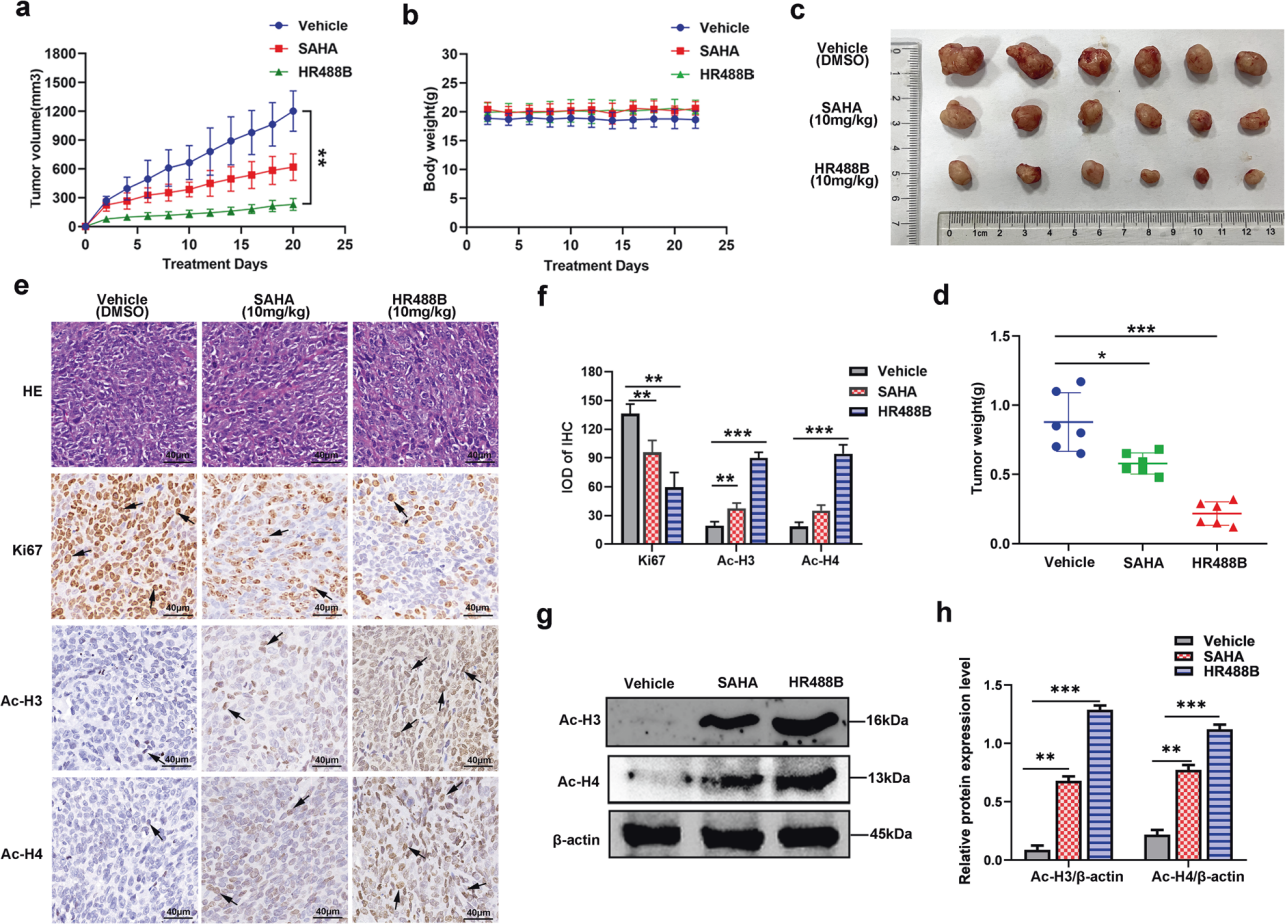
HR488B suppresses tumor progression in CRC xenograft models. HCT116 cells (1 × 10^6^) were injected into BALB/c nude mice, and mice were allocated to six groups after 7 days of tumor-cell implantation. **a** Tumor volume (length × width^2^ × 0.5) was measured every 2 days and treated with vehicle (5% DMSO in PBS, ip, *n* = 6), SAHA (10 mg/kg, ip, *n* = 6) and HR488B (10 mg/kg, ip, *n* = 6). **b** The mouse weight was quantified in each group. **c** The representative stripped images of the tumor entity after being treated with vehicle, SAHA, and HR488B for 2 weeks. **d** The scatter plot summarized the weight of the tumors. **e** Representative hematoxylin and eosin (HE) staining of tumor tissues. And immunocytochemical staining for Ki67, Ac-H3, and Ac-H4 expression in tumor tissues from nude mice (Magnification, 400×. Scale bar, 40 μm). **f** The IHC results were analyzed by Image-Pro Plus 6.0 (*n* = 5 fields of view). **g** Western blot analysis of Ac-H3 and Ac-H4 in tumor tissues and β-actin was detected as the endogenous loading control, accordingly. **h** The statistical result of (**g**). All data are shown as mean ± SD, two-way ANOVA, *p < 0.05, **p < 0.01, ***p < 0.001.

We next validated the anti-tumor effects of HR488B in vivo using CRC xenograft mouse-bearing HCT116 tumors. HCT116 cells were implanted subcutaneously into the dorsal flank of nude mice. After 7 days, mice were injected intraperitoneally with either DMSO, HR488B (10 mg/kg/d), or SAHA (10 mg/kg/d) over 21 days, and the tumor volume and body weight were measured after every 2 days. Our data indicated that HR488B significantly induced a marked reduction in tumor volume compared with the control group and SAHA treatment group (Fig. 3a). However, the body weight of mice did not decrease notably, which meant that the toxicity of these HDACis was tolerable (Fig. 3b). As expected, the average tumor weight in the HR488B-treated group was obviously lower than that in the control and SAHA-treated group (Fig. 3c, d). HR488B treatment of HCT116 xenografts reduced the expression of the proliferative marker Ki67 and increased the expression of the Ac-H3 and Ac-H4 levels (Fig. 3e, f). Western blot analysis of the tumor tissue excised from the HCT116 xenografts mice also confirmed a significant increase of the Ac-H3 and Ac-H4 levels (Fig. 3g, h). Thus, our results showed the HDACs inhibitory effect of HR488B effectively in vivo, thereby greatly shrinking tumors, which were consistent with our in vitro studies observed in CRC cells treated with HR488B and also providing evidence of the potent efficacy of HR488B in CRC therapy.

### HR488B regulates genes involved in cell cycle progression

**Fig. 4:**
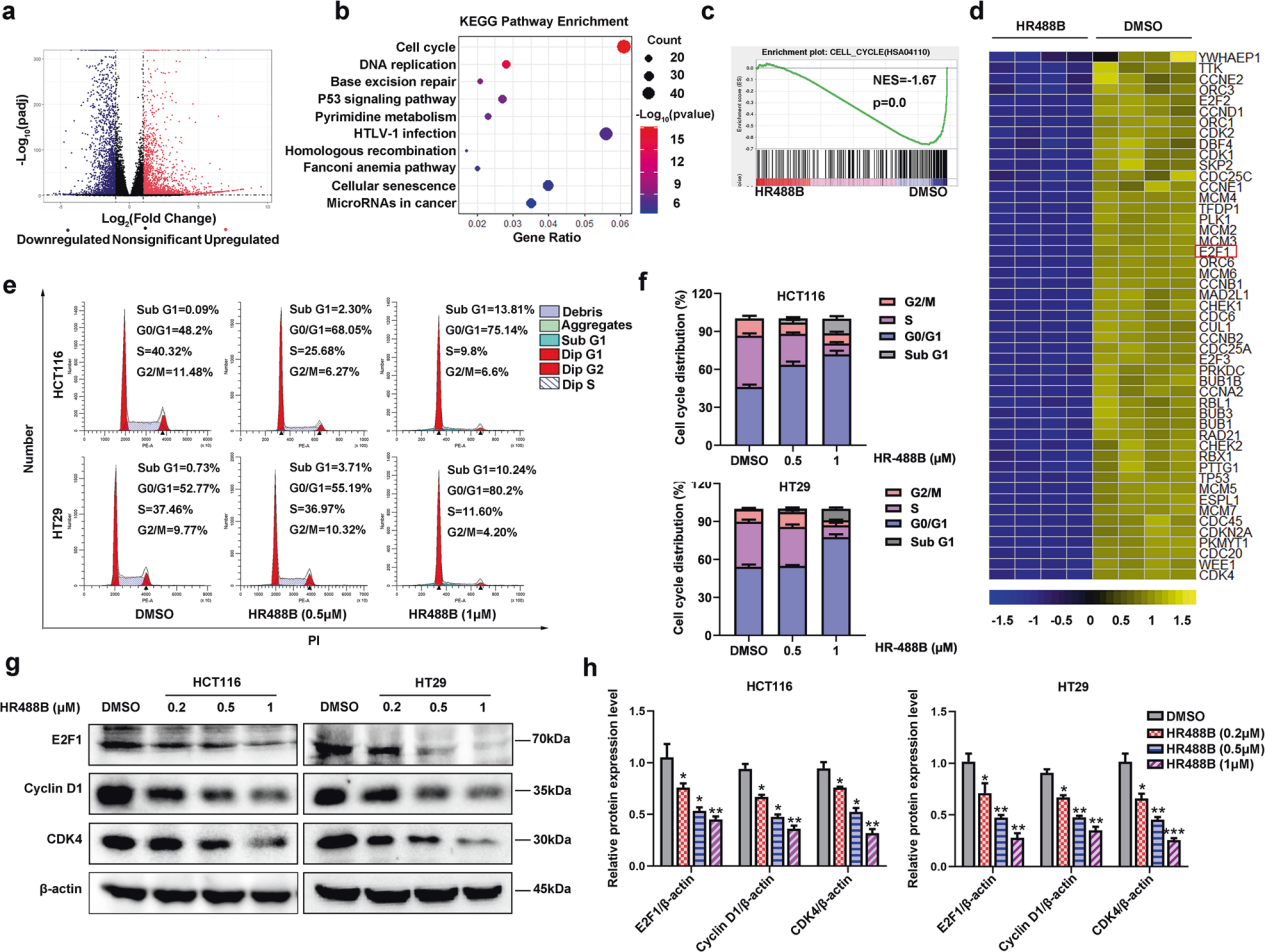
HR488B regulates cell cycle progression in CRC. **a** Volcano plot showed differentially expressed genes induced by HR488B. **b** KEGG Pathway enrichment was analyzed based on the subsets of downregulated DEGs. **c** GSEA analysis of HR488B treatment revealed enrichment of cell cycle. **d** Cell cycle-related genes were significantly suppressed by HR488B. **e** The cell cycle of HCT116 and HT29 treated with DMSO or HR488B (0.5 and 1 μM) for 24 h was examined by flow cytometry analyses. **f** The statistical result of (**e**). **g** HCT116 and HT29 cells were treated with DMSO or various concentrations of HR488B (0.2, 0.5, and 1 μM) for 24 h, respectively, and the expression of E2F1, CDK4, and Cyclin D1 was detected by Western blot analysis, and β-actin was detected as the endogenous loading control, accordingly. **h** The statistical result of (**g**). All data are shown as mean ± SD, *n* = 3, two-way ANOVA, *p < 0.05, **p < 0.01, ***p < 0.001.

To further elucidate the specific molecular mechanisms of the anti-tumor effect of HR488B on CRC, we next performed RNA-Seq to analyze the transcriptomes of HCT116 treated with DMSO or HR488B for 24 h (Fig. 4a–d). HR488B treatment resulted in even stronger changes in HCT116 cell transcription with 3105 upregulated and 2246 downregulated genes. (Figs. 4a, S6). Subsequently, we performed KEGG analysis on the genes that were downregulated by HR488B and found that HR488B treatment significantly downregulated genes associated with cell cycle processes (Fig. 4b). Gene set enrichment analysis (GSEA) also suggested that HR488B suppressed CRC cell growth via cell cycle pathway (Fig. 4c). The expression pattern of important genes regulating cell cycle progression was remarkably suppressed by HR488B treatment compared to DMSO-treated HCT116 cells, including E2F1, CDK4, Cyclin D1, and others (Fig. 4d). It’s worth noting that E2F1 is an important transcription factor of the cell cycle and is implicated in cell growth regulation. Meanwhile, an increase in E2F transcription is often associated with inappropriate cell proliferation [25]. In view of the RNA-Seq results, to take this a step further validated whether or not the cytotoxic effect on CRC cells of HR488B was mediated by the cell cycle pathway. As expected, HR488B treatment significantly induced cell cycle arrest in G0/G1 phase in a dose-dependent manner both in HCT116 and HT29 cells (Fig. 4e, f). Similarly, HR488B treatment decreased the expression levels of cell cycle regulatory proteins E2F1, CDK4, and Cyclin D1 in HCT116 and HT29 cells (Fig. 4g, h). Based on the results above, we considered that HR488B exhibited ideal anti-tumor activity via inducing the cell cycle arrest in CRC cells.

### HR488B enhances mitochondrial dysfunction, ROS generation, and DNA damage accumulation to induce apoptosis in CRC cells

**Fig. 5:**
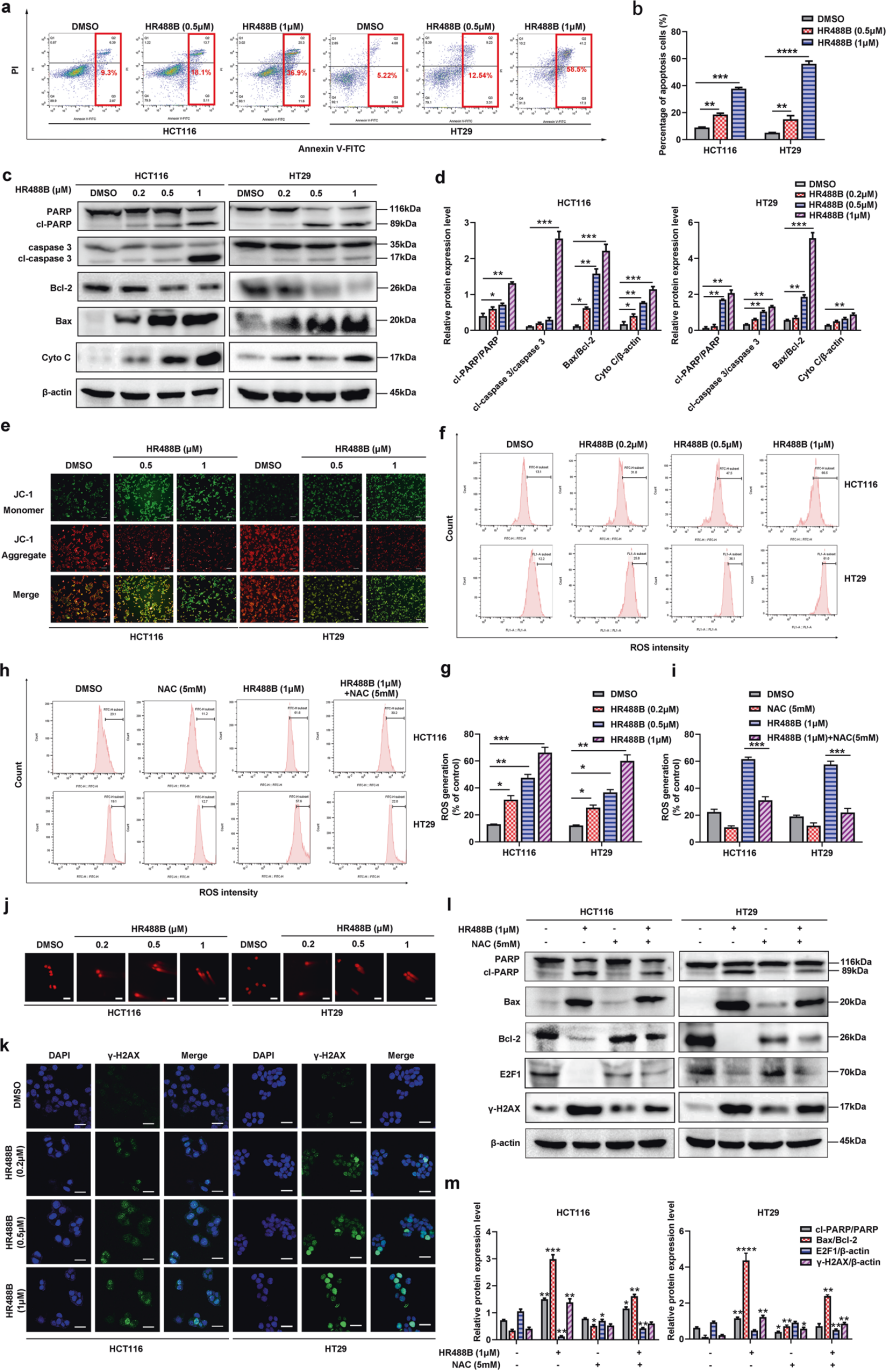
HR488B induces apoptosis in CRC cells via mitochondrial dysfunction, ROS accumulation, and DNA damage. **a** Apoptosis of HCT116 and HT29 cells induced by DMSO or HR488B (0.2, 0.5, and 1 μM) treatment for 48 h were examined by using Annexin V-FITC/PI double-staining analysis. **b** The statistical result of (**a**). **c** HCT116 and HT29 cells were incubated with DMSO or HR488B (0.2, 0.5, and 1 μM) for 24 h. PARP, cl-PARP, caspase 3, cl-caspase 3, Bax, Bcl-2, and Cyto C protein levels were examined by Western blot analysis, and β-actin was detected as the endogenous loading control, accordingly. **d** The statistical result of (**c**). **e** HCT116 and HT29 cells were treated with DMSO or 0.5 and 1 µM of HR488B for 24 h. The treated cells stained with JC-1 were pictured by fluorescence microscope to estimate the alteration in MMP (Scale bar, 100 μm). **f** HCT116 and HT29 cells were treated with DMSO or HR488B (0.2, 0.5, and 1 μM) for 24 h. Representative flow cytometry histograms displaying levels of fluorescent DCFH-DA in cells were presented. **g** Statistical analysis of the percentage of ROS generation (**f**). **h** HCT116 and HT29 cells were pretreated with or without NAC (5 mM) for 2 h, then treated with HR488B (1 μM) and NAC (5 mM) alone or in combination for 24 h. Intracellular ROS was measured by flow cytometry after 10 μM DCFH-DA staining. **i** Statistical analysis of the percentage of ROS generation (**h**). **j** HCT116 and HT29 cells were treated with DMSO or HR488B (0.2, 0.5, and 1 μM) for 24 h on DNA damage detected by alkaline comet assay (Scale bar, 50 μm). **k** HCT116 and HT29 cells were treated with DMSO or HR488B (0.2, 0.5, and 1 μM) for 24 h, and the expression of γ-H2AX was analyzed by immunofluorescence. Green represents the cells stained with anti-γ-H2AX antibody, and blue represents the nuclei stained with DAPI (Scale bar, 25 μm). **l** HCT116 and HT29 cells were pretreated with or without NAC (5 mM) for 2 h, then treated with HR488B (1 μM) and NAC (5 mM) alone or in combination for 24 h. PARP, cl-PARP, Bax, Bcl-2, E2F1, and γ-H2AX protein expression were evaluated by Western blot analysis, and β-actin was detected as the endogenous loading control, accordingly. **m** The statistical result of (**l**). All data are shown as mean ± SD, *n* = 3, two-way ANOVA, *p < 0.05, **p < 0.01, ***p < 0.001, ****p < 0.0001.

Besides cell cycle arrest, it is noteworthy that cell apoptosis is another effective mechanism in the induction of cell death, and E2F1 has also been previously identified as a key regulatory gene of apoptosis [26]. Considering these facts, we further investigated the percentages of HCT116 and HT29 cells undergoing apoptosis after HR488B treatment by flow cytometric analysis after staining with annexin V-FITC. Increased percentages of apoptotic cells were observed in both HCT116 and HT29 cells with increasing concentrations of HR488B at 48 h after treatment (Fig. 5a, b). Concomitantly, the fact that HR488B led to increased apoptosis was also confirmed by Western blot analysis. HR488B dose-dependent increased the expression levels of apoptosis-related protein cleaved-PARP (cl-PARP), cleaved-caspase 3 (cl-caspase 3), and Bax, while it markedly decreased the expression level of Bcl-2 (Fig. 5c, d). In addition, numbers of evidence indicated that mitochondrial dysfunction can orchestrate the apoptotic pathway [27]. Meanwhile, HR488B incubation could increase cytochrome C (Cyto C) release in HCT116 and HT29 cells (Fig. 5c, d). To explore the mechanism of cell apoptosis induced by HR488B, we assayed the effect of HR488B on Mitochondrial Membrane Potential (MMP) by JC-1 staining, which was an early marker of mitochondria-mediated apoptosis. Figure 5e showed that HR488B dose-dependent caused a decrease in red fluorescence, while an increase in green fluorescence compared with control group. These results signified that HR488B could induce mitochondrial dysfunction in CRC cells. It is well established that MMP loss is often associated with reactive ROS generation [28]. Thus, we further analyzed the level of ROS generation by flow cytometry and confirmed that the ROS production was increased dose dependently after HR488B treatment in HCT116 cells and HT29 cells (Fig. 5f, g). Subsequently, to prove the role of ROS in HR488B-induced apoptosis, we pre-incubated CRC cells with the ROS scavenger NAC to determine whether NAC could attenuate apoptosis induced by HR488B. The result displayed that the NAC obviously suppressed the ROS accumulation in HR488B-treated group (Fig. 5h, i). Moreover, compared with the HR488B treatment alone group, both the inhibitory effects on Bcl-2 and the stimulative effects on cl-PARP, cl-caspase 3, and Bax were remarkably diminished in the NAC and HR488B combined treatment group (Fig. 5l, m), which confirmed that HR488B indeed triggered the apoptosis of CRC cells by enhancing ROS levels. In addition, there is evidence demonstrating that DNA damage is induced by ROS in CRC [29]. Therefore, we assessed DNA damage in HR488B-treated CRC cells using alkaline comet assay. As expected, a significant increase in DNA damage was observed in HCT116 cells and HT29 cells after HR488B treatment (Fig. 5j). γH2AX is widely acknowledged as a DNA double-strand break marker and the accumulation of γH2AX reflects the extent of DNA damage [30]. Accordingly, the nuclear γH2AX levels were increased by HR488B treatment (Fig. 5k). Meanwhile, the increase in γH2AX protein expression level induced by HR488B also was proved to be attenuated by NAC (Fig. 5l, m). Taken together, these data indicated that HR488B-induced CRC cells apoptosis was caused by mitochondrial dysfunction, excessive ROS accumulation, and DNA damage.

### HR488B-induced inhibition of CRC through attenuating E2F1/Rb/HDAC1 complex dissociation

**Fig. 6:**
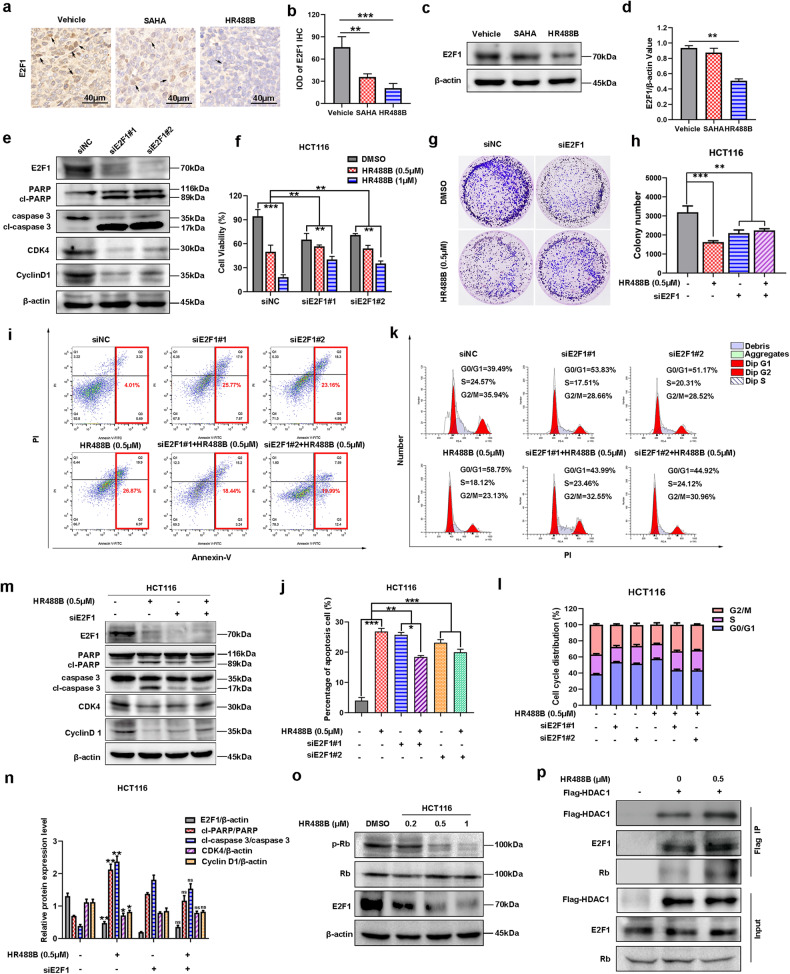
HR488B-induced inhibition of CRC through attenuating E2F1/Rb/HDAC1 complex dissociation. **a** HCT116 cells were injected into nude mice after 7 days and the mice were treated with vehicle (5% DMSO in PBS), SAHA (10 mg/kg), or HR488B (10 mg/kg) with i.p. for 3 weeks. Representative IHC image showing that HR488B decreased the expression of E2F1 in vivo (Magnification, 400×. Scale bar, 40 μm). **b** The IHC results were analyzed by Image-Pro Plus 6.0 (*n* = 5 fields of view). **c** HCT116 xenografts treated with vehicle, SAHA, or HR488B were isolated to detect expression of E2F1 by Western blot analysis, and β-actin was detected as the endogenous loading control, accordingly. **d** The statistical result of (**c**). **e** HCT116 cells were transfected with siRNAs targeting E2F1 (siE2F1#1 and siE2F1#2) or siNC for 24 h. Western blot analysis was performed to measure the expression of E2F1, PARP, cl-PARP, caspase 3, cl-caspase 3, CDK4, and Cyclin D1, and β-actin was detected as the endogenous loading control, accordingly. **f** HCT116 cells were transfected with siRNAs targeting E2F1 (siE2F1#1 and siE2F1#2) or siNC, and 24 h later, cells were treated with or without HR488B (0.5 and 1 μM) for 24 h. Cell viability was assessed with the CCK-8 assay. **g** HCT116 cells were transfected with siRNAs targeting siE2F1 or siNC, and 24 h later, colony-forming abilities of cells after treating cells with DMSO or HR488B (0.5 μM) for 14 days. **h** The statistical result of (**g**). **i** HCT116 cells were transfected with siRNAs targeting E2F1 (siE2F1#1 and siE2F1#2) or siNC, 24 h later, cells were treated with DMSO or HR488B (0.5 μM) for 48 h, and apoptosis was detected by Annexin V-FITC/PI double-staining analysis. **j** The statistical result of (**i**). **k** HCT116 cells were transfected with siRNAs targeting E2F1 (siE2F1#1 and siE2F1#2) or siNC, 24 h later, cells were treated with DMSO or HR488B (0.5 μM) for 24 h, and cell cycle distribution was assessed by flow cytometry. **l** The ratio of G1, S, and G2/M phases was shown in histography (**k**). **m** HCT116 cells were transfected with siRNAs targeting siE2F1 or siNC, and 24 h later, cells were treated with DMSO or HR488B (0.5 μM) for 24 h, and the expression of E2F1, PARP, cl-PARP, caspase 3, cl-caspase 3, CDK4, and Cyclin D1 protein was determined by Western blot analysis, and β-actin was detected as the endogenous loading control, accordingly. **n** The statistical result of (**m**). **o** HCT116 cells were treated with DMSO or HR488B (0.2, 0.5, and 1 μM) for 24 h, respectively, and the expression of Rb, p-Rb, and E2F1 protein was detected by Western blot analysis, and β-actin was detected as the endogenous loading control, accordingly. **p** 293T cells were transfected with the flag-HDAC1 plasmid for 12 h, and cells were then treated with HR488B (0.5 μM) for 24 h; HDAC1 was immunoprecipitated using an anti-Flag antibody and the expression of endogenous E2F1 and Rb was detected by Western blot analysis. All data are shown as mean ± SD, *n* = 3, two-way ANOVA, *p < 0.05, **p < 0.01, ***p < 0.001.

As mentioned above, E2F1 expression was markedly downregulated by HR488B in CRC cells. Multiple studies have documented that the E2F/Rb complex associated with HDAC at E2F-responsive promoters repressed transcription of partially proliferation genes and halt the cell cycle progression [31, 32]. This observation raised an interesting possibility that decreasing the dissociation of the E2F1/Rb/HDAC1 complex maybe repress the transcription and proteasomal degradation of E2F1, leading to the suppression of the E2F1 function in solid tumors. According to the above findings, we next sought to clarify to explore whether the mechanisms underlying HR488B-inhibited development of CRC are related to E2F1/Rb/HDAC1 complex. We firstly detected HR488B markedly downregulated E2F1 expression in an HCT116 xenograft tumor tissue by IHC staining (Fig. 6a, b). Meanwhile, Western blot analysis of these tumor tissues confirmed that HR488B indeed effectively suppressed E2F1 protein expression in vivo (Fig. 6c, d), which was consistent with the results of in vitro studies. To elucidate the potential role of E2F1 in HR488B-induced CRC inhibition, we subsequently performed E2F1 knockdown in HCT116 cells. As shown in Fig. 6e, E2F1 knockdown significantly increased the expression of apoptosis markers cl-PARP and cl-caspase 3, while remarkably downregulating the expression of cell cycle-related proteins CDK4 and Cyclin D1, which confirmed the essential role of E2F1 in cell cycle and apoptosis. Furthermore, E2F1-knockdown cells were less sensitive to HR488B-induced inhibition of HCT116 cell proliferation and colony formation compared with the siNC cells (Fig. 6f–h). As shown in Fig. S7, overexpression of HDAC1 or E2F1 partially reversed the inhibitory effect of HR488B on cell viability and the co-expression of HDAC1 and E2F1 was more effective to reverse the inhibitory effect of HR488B, which confirmed that HR488B executed its anti-cancer effect via the HDAC1-E2F1 axis. We also found that the knockdown of E2F1 blocked the HR488B-induced accumulation of ROS in HCT116 cells (Fig. S8a, b). In addition, compared with the siNC group, the apoptotic rate and the number of G0/G1 phase HCT116 cells induced by HR488B could not be further reduced in the siE2F1 group (Fig. 6i–l). In support of this notion, HR488B treatment did not obviously affect the protein expression levels of cl-PARP, cl-caspase 3, CDK4, and Cyclin D1 in E2F1-knocking cells (Fig. 6m, n). Taken together, our results indicated that E2F1 was required for HR488B-induced CRC inhibition. As reported, phosphorylation of Rb can destabilize the set of E2F1/Rb/HDAC1 interactions resulting in the dissociation of the complex [16]. Figure 6o showed that HR488B significantly inhibited the phosphorylation of Rb in HCT116 cells, accompanied by downregulation of E2F1 expression protein. Therefore, we speculate that HR488B could prevent E2F1 release from the E2F1/Rb/HDAC1 complex by reducing the phosphorylation level of Rb, thereby affecting the E2F1 function. To formalize our speculation, we performed co-immunoprecipitation of HDAC1 in 293T cells. The result of co-immunoprecipitation provided further evidence that HR488B indeed promoted bonding ability of HDAC1 with E2F1 protein and Rb protein, promoting the E2F1/Rb/HDAC1 complex formation (Fig. 6p). The above results demonstrated that HR488B could inhibit the malignant behavior of CRC cells by targeting E2F1/Rb/HDAC1 complex.

## Discussion

**Fig. 7:**
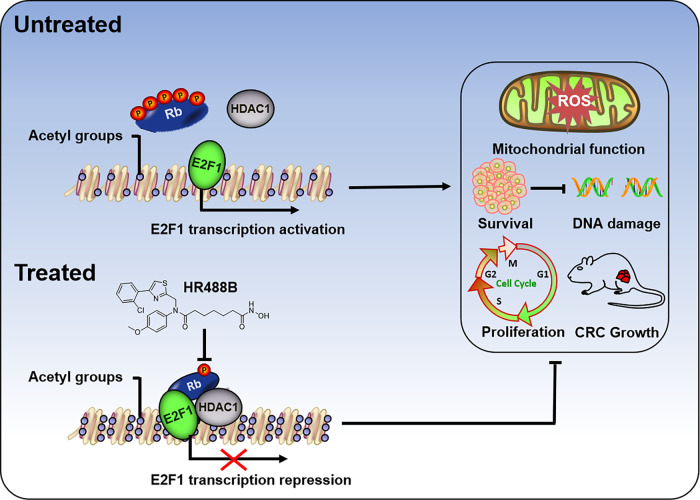
Schematic illustration of the role of HR488B in CRC. HR488B, a novel inhibitor of HDACs, potently inhibits CRC growth in vitro and in xenografts. Mechanistically, HR488B attenuates E2F1/Rb/HDAC1 complex dissociation by decreasing the phosphorylation of the Rb protein, which regulates the E2F1 transcription activity. The reduction in E2F1 expression influences mitochondrial function and ROS levels. HR488B treatment induces mitochondrial dysfunction, excessive accumulation of ROS levels, and DNA damage, resulting in cell cycle arrest and cell apoptosis, eventually inhibiting CRC development.

Despite advances in surgical techniques and the promotion of early screening improved the survival rate of CRC patients in recent years, approximately 900,000 still die annually, and the 5-year survival rate of patients with advanced-stage tumors or metastasis is even less than 20% [2, 33, 34]. Our work presented here identified a novel HDACi HR488B that effectively inhibited CRC development and supported the future evaluation of HR488B as a powerful candidate for CRC therapy. Further mechanistic studies revealed that HR488B could reduce the E2F1/Rb/HDAC1 complex dissociation by inhibiting the phosphorylation of Rb protein, suppressing the protein expression of the free E2F1 transcription factor, and ultimately inducing cell cycle arrest, apoptosis, mitochondrial dysfunction, and DNA damage (Fig. 7).

Previous work suggests that HDACis can inhibit CRC growth, yet the efficacy, durability, and specificity of these treatments need to be further improved [3]. Therefore, developing the novel HDACi with stronger inhibitory activity of cancers and exploring its molecular mechanisms in the regulation of tumors are of paramount importance. In this study, we identified HR488B as a new HDACi that displayed potent anti-CRC activity both in vitro and in vivo (Figs. 1, 3). HR488B was a thiazole-containing hydroxamic acid small molecule compound that obtained more excellent HDACs inhibitory effects and more significant anti-proliferative activities compared with SAHA, an FDA-approved HDACi (Figs. S1–3). Especially, HR488B exhibited superior selectivity for HDAC1 over HDAC2, HDAC6, and HDAC8 (Table 1). The high potency and selectivity of HR488B were further confirmed and rationalized by the molecular docking, molecular dynamics simulations, and MTS analysis. We found that HR488B was directly bound to HDAC1 with a more stable state and stronger binding affinity in vitro. The HR488B binding with HDAC1 protein could preliminarily explain the better inhibitory activity of HR488B than SAHA and HDAC2 (Figs. 2 and S4, 5). Furthermore, in siHDAC1 HCT116 cells, the inhibition of cell viability and increase in the expression of Ac-H3 and Ac-H4 proteins induced by HR488B were significantly reduced, indicating that HR488B exerted its anti-CRC activity through HDAC1 (Fig. 2). These results collectively confirm that HR488B is a potent inhibitor of HDAC1.

To further explore the molecular mechanisms underlying the biological role of HR488B against CRC, we performed RNA-Seq analysis (Figs. 4 and S6). There was a significant enrichment of the cell cycle within the set of HR488B-repressed genes in CRC cells. Flow cytometry and Western blot analysis confirmed that HR488B induced G0/G1 cell cycle arrest and downregulated the expression levels of cell cycle checkpoint proteins Cyclin D1 and CDK4. Besides, HR488B markedly decreased the important cell cycle and apoptosis regulator E2F1 expression (Fig. 4). HR488B decreased the transcriptional level of E2F1 in HCT116 cells (Fig. S9a), but did not affect its protein stability, as shown by the CHX assay (Fig. S9b). E2F1 is usually elevated in CRC tissues compared with normal tissues, which is associated with a worse prognosis [35]. However, both the pro-proliferate and pro-apoptotic properties of E2F1 are still controversial in CRC. Considering that the pro-apoptotic effect of E2F1 depended predominantly on exogenous administration of E2F1 in vitro, and unfavorable survival in the patients with CRC who exhibited increased expression of E2F1, we speculate that the pro-proliferate effect of endogenous E2F1 might be dominant [11, 36]. Recently, a possible mechanism has been reported that different modifications of E2F1 protein could activate different downstream genes thereby carrying out divergent functions in CRC, which was further verified by a report of a similar mechanism in prostate cancer [37, 38]. Therefore, the complex behaviors of E2F1 in HR488B-induced inhibition of CRC deserve further study.

Mitochondria, as hubs of bioenergy, biosynthesis, and signaling transduction, regulate ROS production and initiation of apoptosis [39]. In the process of cell apoptosis mediated by external factors, increased mitochondrial membrane permeability could result in a decrease in MMP followed by the release of apoptotic proteins from mitochondria into the cytoplasm, finally triggering caspase-dependent or caspase-independent apoptotic pathways [40, 41]. Our results indicated that HR488B remarkably decreased MMP to release Cyto C, which further activated caspase 3 downstream to cleave PARP, thereby inducing CRC cell apoptosis. Notably, there was a reciprocal regulatory role between E2F1 and mitochondria, in which E2F1 could regulate mitochondrial functions via repressing the expression of key genes implicated in the respiratory chain, tricarboxylic acid (TCA) cycle, uncoupling respiration, and transcription regulation. Moreover, E2F1 expression was also sensitive to mitochondrial ROS levels [25, 42]. Previous studies have uncovered excessive ROS generated in response to external stimuli could be responsible for the accumulated damage to DNA [43]. In addition, Eui-Hwan Choi and Keun Pil Kim found that E2F1, as a key regulator of DNA damage, depletion resulted in the accumulation of DNA damage by downregulating the levels of homologous recombination to interrupt DNA replication and reducing DNA repair mediated by RAD51 [44]. Based on these backgrounds, we investigated the effects of HR488B on mitochondria dysfunction, ROS levels, and DNA damage. HR488B significantly caused apoptosis by mitochondrial dysfunction, DNA damage, and ROS generation accumulation mediated by E2F1 downregulation (Fig. 5). The results of siE2F1 further confirmed that E2F1 was essential for HR488B-induced CRC suppression (Figs. 6 and S8).

E2F1 activity is tightly controlled via its interactions with different regulators among which Rb and HDAC1 are critical. Studies reported that the Rb binding to E2F1 and HDAC1 suppressed the transcription of E2F1, while Rb phosphorylation destabilized the overall E2F1/Rb/HDAC1 complex remarkedly [31, 45]. The regulation of intracellular histone deacetylation and acetylation through HDACs can control the E2F1 transcription levels [31]. Our study found that HR488B could prevent E2F1 from releasing from E2F1/Rb/HDAC1 complex by inhibiting the phosphorylation of Rb protein, providing a theoretical basis for HR488B treating CRC patients with high expression of E2F1 (Fig. 6). In addition, in the G1 phase, the CDK4-Cyclin D1 complex is known to phosphorylate and inactivate Rb protein [46]. We knocked down CDK4 in HCT116 cells and found that siCDK4 blocked the inhibitory effect of HR488B on Rb phosphorylation, indicating that HR488B inhibited Rb phosphorylation via CDK4 (Fig. S10). Furthermore, inhibition of Rb phosphorylation in solid tumors has also been reported with the marketed HDACis, which further indicates that HR488B has great potential to be a novel therapeutic agent for CRC. Moreover, previous studies have reported that inhibition of acetylation levels could lead to a decrease in the amount and promoter recruitment of the E2F1 transcription factor, influencing the expression of the downstream cell cycle, apoptosis, and DNA repair-related genes [47, 48]. Thus, we believe that HR488B may regulate the recruitment of E2F1 promoters by affecting the combined state of HDAC1 and E2F1 promoters, which needs to be further explored. On the other hand, Li et al. identified a novel positive regulator, Nemo-like kinase, that dissociated HDAC1 from the E2F1 complex to promote the transcription of E2F1 [49]. Recent research showed that HDGF-related protein 3 was directly bound to the E2F1 promoter to contribute to the interaction of HDAC1 and histone around the E2F1 promoter [50]. These studies demonstrated that there were other proteins in the E2F1/Rb/HDAC1 complex that were not fully understood in regulating the expression of E2F1, which need to be further identified.

Furthermore, drug resistance is a common and challenging phenomenon associated with many anti-cancer drugs. After prolonged usage in patients, SAHA has been documented to develop drug resistance. This resistance is attributed to multiple factors, including the amplification of intracellular repair mechanisms, changes in epigenetic modifications, and adjustments in cell signaling pathways [5, 51, 52]. In light of this, it is imperative to conduct comprehensive and thorough research to determine whether HR488B exhibits drug resistance following extended periods of treatment and there will be a concerted effort to delve deeper into unraveling the fundamental mechanism involved and to investigate synergistic therapeutic approaches aimed at surmounting the potential drug resistance associated with HR488B.

In summary, our finding that HR488B as a novel HDAC1 inhibitor, that remarkably repressed the growth of CRC both in vitro and in vivo without causing any significant toxicity, and further explored its molecular mechanism. The present study demonstrated that HR488B induced cell apoptosis and cell cycle G0/G1 arrest via downregulating E2F1 expression, which reflected that E2F1 was a key molecule in the anti-CRC role of HR488B. The results of this study revealed that HR488B decreased the expression of E2F1 by attenuating the dissociation of the E2F1/Rb/HDAC1 complex mediated by decreasing the phosphorylation of Rb protein, thereby inhibiting CRC development. In view of E2F1 is associated with poor prognosis of CRC patients, HR488B treatment might contribute to improving the prognosis of patients. Taken together, our study indicated that HR488B might be an efficacious anti-cancer agent for the treatment of CRC.

## Materials and methods

### Synthesis of HR488B

The route adopted for the preparation of HR488B is depicted in Fig. S1. Intermediate (**2**) was constructed from p-methoxy aniline and Bromo acetonitrile, which was coupled with heptane dioic anhydride to yield acid (**4**). Then corresponding ester (**5**) was prepared by esterification of those acids. Subsequently, it was reacted with ammonium sulfide and triethylamine by heating to produce an intermediate (**6**) and then cyclized with 2,2’-dichloroacetophenone to form an intermediate (**8**). Finally, treatment of methyl ester with hydroxylamine to afford the target compound HR488B (≥99% purity). HPLC (Agilent Technologies 1260 Series) was employed for the purity determination.

### Cell lines and cell culture

The HCT116, HT29, H1299, A549, MCF-7, HepG2, and 293T cell lines were obtained from the American Type Culture Collection (ATCC). All cell lines were routinely tested to confirm that they were free of *Mycoplasma*. HCT116, H1299, and 293T cell lines were cultured in RPMI-1640 (Gibco, USA), whereas HT29, A549, MCF-7, and HepG2 cell lines were cultured in DMEM (Gibco, USA). All culture media were supplemented with 10% FBS with 100 U/ml penicillin and 100 μg/ml streptomycin, and cells were cultured at 37 °C in 5% CO_2_. Cells were kept at low passages (3–5 passages) once obtained from vendors.

### Antibodies and reagents

Antibodies against the following proteins were used with source and dilution ratios indicated: Ac-H3 (Beyotime, #AF5620, 1:1000); Ac-H4 (Beyotime, #AF5629, 1:1000); E2F1 (Santa, #sc-251, 1:1000); Cyclin D1 (CST, #2978, 1:1000); CDK4 (CST, #12790, 1:1000); PARP (CST, #9542, 1:1000); cleaved-PARP (cl-PARP) (CST, #5625, 1:1000); caspase 3 (CST, #9662, 1:1000); cleaved-caspase 3 (cl-caspase 3) (CST, #9661, 1:1000); Bcl-2 (CST, #4223, 1:1000); Bax (CST, #5023, 1:1000); Cyto C (Beyotime, #AF2047, 1:1000); γ-H2AX (CST, #9718, 1:1000); HDAC1 (CST, #5356, 1:1000); β-actin (CST, #3700, 1:10,000); Anti-rabbit lgG Fab2 (Sigma, #A0545, 1:10,000); Anti-mouse lgG Fab2 (Sigma, #A4416, 1:10,000). Phosphate-buffered saline (PBS) washing buffer, Fetal bovine serum (FBS), Trypsin-EDTA solution, and Penicillin-Streptomycin solution (PS) (100×) were all purchased from Gibco (Carlsbad, CA, United States). Cell Counting Kit-8 (CCK-8), RIPA lysis buffer, crystal violet, and ROS assay kit were purchased from Beyotime (Shanghai, China). N-Acetyl-L-cysteine (NAC) was purchased from MedChemExpress (Shanghai, China). Bicinchoninic acid (BCA) Protein assay kit was acquired from TIANGEN (Shanghai, China). The cocktail was obtained from Roche (Basel, Lewes, UK). Polyvinylidene fluoride (PVDF) membranes were purchased from Millipore (Billerica, MA, United States). Protein A/G agarose was purchased from Santa Cruz Biotechnology (CA, United States). pcDNA3.1-Flag-HDAC1 plasmids were obtained from Hunan Fenghui Biotechnology Co., Ltd (Changsha, Hunan, China). pcDNA3.1-HA-E2F1 plasmids were obtained from Hubei Miaoling Biotechnology Co., Ltd (Wuhan, Hubei, China). HDAC1 protein (ab101661, Abcam, Cambridge, UK). The Monolith NT™ Protein Labeling Kit Blue (L003, NanoTemper Technologies GmbH, Munich, Germany). The Reverse Transcription kit (KR116, TIANGEN, China). SYBR® Green Real-time PCR Master Mix (KR123, TIANGEN, China). SAHA (≥99% purity) was donated by the Feifei Yang University of Jinan. All other chemicals were determined to be of high purity and were purchased from commercial sources.

### Cell viability assay

The cells were cultured in 96-well plates at a density of 5 × 10^3^ cells/well. Cell viability was assessed with CCK-8 Kit at indicated time post-treatment according to the manufacturer’s instructions. To estimate the viability of the cells, the absorbance of 450 nm (OD450) was measured with a Microplate Reader (BIO-TEK, Inc., Winooski, VT, United States). The IC_50_ value was calculated by GraphPad Prism 8.0 (San Diego, CA, United States) software.

### Western blot analysis

Western blot analysis was performed as in our previous reports [53]. The cells were lysed in RIPA buffer with a complete protease inhibitor cocktail for 10 min on ice followed by centrifugation at 4 °C. The concentration of proteins was determined by the BCA Protein Kit (TIANGEN, Shanghai, China). Equal amounts of total proteins were resuspended in loading buffer, boiled at 100 °C for 5 min, and separated by 10–15% sodium salt-polyacrylamide gel electrophoresis (SDS-PAGE). Proteins were transferred onto PVDF membranes (Merck Millipore, #IPFL00010, Germany), then the membranes were blocked with 5% non-fat dry milk, and the membranes were incubated with indicated primary antibodies diluted in BSA buffer, overnight at 4 °C. The membranes were washed three times in TBST followed by 1 h incubation with secondary antibodies conjugated with horseradish peroxidase (HRP) at room temperature. Then the membranes were washed before enhanced chemiluminescence. Immunoblots were visualized with the Bio-Rad ChemiDoc XRS system. Quantification was directly performed on the blot using the Image Lab software.

### Colony formation assay

Colony formation assay was used to examine the long-term effects of HR488B on CRC cell growth. Cells were seeded onto 6-well plates at 1000 cells/well, and after 24 h, cells were treated with different concentrations of HR488B. The culture medium was refreshed every other day. Cells were then continuously incubated in a new fresh medium for 10–14 days, the colonies were fixed with methanol for 15 min, stained with 0.5% crystal violet for 15 min, and photographed. Colonies were manually counted using ImageJ software.

### HDAC isoforms activity assay

The HDAC inhibition assay for HR488B was conducted by Reaction Biology Corporation. HR488B was suspended in a 10 mM DMSO stock solution and tested in singlet 10-dose inhibitory concentration (IC_50_) mode with a 3-fold serial dilution starting at 1 µM against HDACs. The fluorescence detection method was adopted using Ac-Lys-Tyr-Lys (Ac)-AMC substrates. The IC_50_ values were calculated using GraphPad Prism software (La Jolla, CA, USA).

### Molecular docking and molecular dynamics simulation

Molecular docking study was carried out by using AutoDock 4.2.6 [54]. The crystal structure of human HDAC1 (PDB ID: 4BKX) retrieved from Protein Data Bank (http://www.pdb.org) was used for molecular docking. To prepare the protein for docking, AutoDock Tools was applied to remove water and co-crystallized ligands from the crystal structure and add polar hydrogens and Kollman charges [55]. The 3D structures of SAHA and HR488B were built with Chemoffice 2019. The grid box center was set as coordinates of x, y, z = −49.617, 18.378, −4.783, and the grid size was 28.5 Å × 24.75 Å × 24.75 Å. The other parameters for AutoDock 4.2.6 were set as default. The binding interaction of the protein-ligands complex has been observed by using UCSF Chimera 1.16 and BIOVIA Discovery Studio Visualizer v21.1.0.20298 (BIOVIA). The lowest energy conformation was selected for molecular dynamics (MD) simulation analysis. All MD simulations were performed using the GROMACS (2020.6). The topology for the HDAC1-HR488B complex was prepared using Amber99 and GAFF force fields, respectively [56]. All molecules have been solvated into the water environment and added ions. HDAC1-HR488B complex was performed the energy minimization to ensure no steric clashes of the system. Before the 10 ns MD simulation, we performed NVT and NPT equilibration. The MD simulation of the HDAC1-HR488B complex was performed at 10 ns with a time step of 2 fs. Finally, we analyzed the MD simulation results.

### Microscale thermophoresis (MST) analysis

MST was performed with human recombinant HDAC1 and HR488B. HDAC1 was fluorescently labeled with the Monolith NT™ Protein Labeling Kit Blue according to the manufacturer’s instructions. The Monolith NT.115 system was used with Monolith NT.115 standard capillaries. The MST with HR488B was performed with 99% LED power, 20% MST power, and a final protein concentration of 200 nM. The NanoTemper Analysis Software was used to fit the data based on the law of mass and to calculate the dissociation constant Kd for each ligand.

### Transfection of siRNAs

HCT116 were transfected with siRNA oligonucleotides using Lipo8000™ Transfection Reagent (Beyotime, Shanghai, China). siRNAs were synthesized in GenePharma Co., Ltd (Shanghai, China), and 24 h after transfection, the cells were used for subsequent experiments. The sequences of siRNAs against E2F1 were 5’-CUGCAGAGCAGAUGGUUAUTT-3’ and 5’-GACCACCUGAUGAAUAUCUTT-3’, siHDAC1 were 5’-GCUCCUCUGACAAACGAAUTT-3’ and 5’-CCGGUCAUGUCCAAAGUAATT-3’, and siCDK4 was 5’-CUCUUAUCUACAUAAGGAU-3’.

### Animal experiments

Male BALB/c nude mice (5–6 weeks) were purchased from the Jiesijie Experimental Animal Co. Ltd (Shanghai, China). All mice were maintained under SPF conditions and at constant temperature (25 °C) and relative humidity (65%) with a 12 h light/dark cycle. HCT116 cells (1 × 10^6^) were inoculated into 6–7 weeks-old mice subcutaneously. Tumor volumes were evaluated by calipers and calculated using the standard formula: volume = length × width^2^ × 0.5. Once tumor volume exceeded approximately 100 mm^3^. Mice were randomly separated into three groups (six mice per treatment group) and received intraperitoneal injections of HR488B and SAHA, and the control group was injected with DMSO every 2 days for 3 weeks, respectively. Tumor volume was measured every 2 days. The protocols for animal care and euthanasia were approved by the Institutional Animal Care and Use Committee of Shanghai Ocean University (Shanghai, China).

### Immunohistochemistry

Immunohistochemical staining was performed by Shanghai RecordBio Co., Ltd. (Shanghai, China). Tumor sections were immunostained with specific anti-Ac-H3, anti-Ac-H4, anti-E2F1, and anti-Ki67 antibodies. The images were captured using a Pannoramic MIDI scanner and analyzed by using Image-Pro Plus 6.0.

### RNA sequencing analysis

HCT116 cells were treated with DMSO or HR488B (1.0 μM), respectively. After 24 h, cells were collected to obtain total RNA using the Total RNA Extraction Kit (Solarbio, Beijing, China) according to the manufacturer’s manual. RNA quantity and quality were assessed on an Agilent 2100 Bioanalyzer. A total of 500 ng of RNA was used to prepare libraries using the NEBNext**®** Ultra RNA Library Perp Kit for Illumina**®**. RNA library sequencing was performed on Illumina NovaSeq 6000 (Illumina, USA) by NovoMagic Co., Ltd. (Beijing, China). Differentially expressed genes (DEGs) in the DMSO group vs the HR488B group were identified based on a |log_2_FC| > 1.0 and an adjusted *P* < 0.05. DEGs with a log_2_FC < 1.0 were considered downregulated genes, while DEGs with a log_2_FC > 1.0 were considered upregulated genes.

### Apoptosis and cell cycle assay

Cell apoptosis was analyzed by an Annexin V-FITC Apoptosis Detection Kit (Beyotime, Shanghai, China). The cell cycle was analyzed by a PI Cell Cycle and Apoptosis Analysis Kit (Beyotime, Shanghai, China). The CRC cells were seeded in 6-well plates at a density of 2 × 10^5^ cells per well and were treated with indicated different doses of HR488B for 24 h or 48 h; DMSO served as vehicle control. After treatment, the cells were collected and stained with Annexin V-FITC and PI following the manufacturer’s protocol to analyze cell apoptosis and cell cycle. Cell apoptosis and the cell cycle were detected by BD FACS Celesta flow cytometry (New York, USA). Apoptosis data were analyzed by FlowJo software, and cell cycle data were analyzed with Modfit LT 4.1 software.

### Mitochondrial membrane potential (MMP) assay

Changes of MMP were investigated using JC-1 Assay Kit (Beyotime, Shanghai, China). HCT116 and HT29 cells were seeded onto 6-well plates at a density of 1 × 10^5^ cells per well and treated with indicated different doses of HR488B for 18 h, DMSO served as vehicle control. According to the manufacturer’s instructions, the treated cells were incubated with JC-1 staining solution in a dark for 20 min at 37 °C and washed cells with dilution buffer. Then the cellular fluorescence of both JC-1 monomers and aggregates was visualized under fluorescent microscopy (Leica DMI8, Germany).

### Reactive oxygen species (ROS) measurement

2,7-Dichlorodihydrofluorescein diacetate (DCFH-DA) was used as ROS probe to detect ROS. CRC cells were seeded onto 6-well plates at a density of 1 × 10^5^ cells per well and treated with indicated doses of HR488B, NAC (5 mM) alone or co-treated for 24 h, DMSO served as vehicle control. Then the cells were harvested, centrifuged, and incubated with 10 μM DCFH-DA at 37 °C for 30 min. Subsequently, the cells were washed twice with PBS. Finally, the mean fluorescence intensity was analyzed using the FACSCelesta flow cytometer (BD Biosciences, San Jose, CA, United States) and data then were evaluated with FlowJo V10 software.

### Immunofluorescence assay

An immunofluorescence assay was performed as previously described [57]. The cells were plated on glass coverslips in 6-well plates and treated with the appropriate concentration of HR488B for 24 h. Cells were washed with PBS and fixed in 4% formaldehyde and then incubated with a γH2AX antibody overnight at 4 °C. The slides were washed 3 times with PBS and stained for 30 min at room temperature with Goat anti-Rabbit IgG (1:500, Invitrogen, Waltham, MA, USA). After staining, nuclei were counterstained with DAPI (Invitrogen, Waltham, MA, USA). Images were taken using a confocal laser scanning microscope (Leica SP8, Germany).

### Alkaline comet assay

CRC cells were subjected to alkaline comet assay, The cells were treated with the appropriate concentration of HR488B for 24 h. Cells collected with PBS were mixed with low melting point agarose gel, spread onto chamber slides, and allowed to cool. Slides were immersed in Lysis Solution and incubated at 4 °C for 1 h. Slides were incubated for 20 min at room temperature (RT) in an Alkaline Unwinding solution (200 mM NaOH, 1 mM EDTA). Slides were submerged in Alkaline Electrophoresis Solution (200 mM NaOH, 1 mM EDTA) inside the electrophoresis chamber, and subjected to 21 volts for 1 h. After electrophoresis, the slides were washed at least three times with 0.4 mM Tris-HCl (pH = 7.5) before staining with propidium iodide (PI) (20 μL) for 10 min. Comet tails were observed with an inverted biological microscope.

### Co-immunoprecipitation (Co-IP)

Co-IP was performed following our previous research [58]. The pc-DNA3.1 HDAC1 plasmids were transfected into 293T cells using Lipofectamine 8000 reagent according to the manufacturer’s instructions. In brief, the plasmids and Lipofectamine 8000 were separately incubated with RPMI-1640 medium for 5 min at RT, after which these two reagents were mixed, incubated at RT for 15 min, and added into 293 T cells, 24 h later, cells were treated with or without HR488B (0.5 μM) for 24 h. 293 T cells were harvested, washed, and resuspended in lysis buffer (50 mM Tris-HCl (pH 7.4); 150 mM NaCl; 1 mM EDTA; 0.5% NP-40; 10% glycerol; protease inhibitor cocktail) and kept on ice for 40 min. The cell extracts were clarified by centrifugation, and proteins were immobilized by binding to anti-Flag M2 affinity gel for 4 h or overnight at 4 °C. The agarose beads were rinsed three times with PBS containing a cocktail of proteinase inhibitors. The immunoprecipitated proteins were then subjected to Western blot analysis.

### Quantitative RT-PCR (qRT-PCR)

After being treated with 0, 0.2, 0.5, and 1 µM HR488B for 24 h, HCT116 cells were collected for RNA extraction. RNA samples were then reverse transcribed into cDNA using the RT Master Mix from the Reverse Transcription kit. Quantitative analysis of target genes was conducted in triplicate using SYBR® Green Real-time PCR Master Mix with 7500 Real-Time PCR System (Thermo Fisher Scientific, Invitrogen, Waltham, MA, USA). The relative expression of each targeted gene was calculated and normalized using the 2−∆∆Ct method relative to reduced glyceraldehyde phosphate dehydrogenase (GAPDH). The sequences of the primers used for qRT-PCR were as follows: GAPDH forward and reverse primers: 5’-CATATGGGGAAGGTGAAGGTCGGAGTC-3’ and 5’-GAATTCTTACTCCTTGGAGGCCATGTGG-3’; E2F1 forward and reverse primers: 5’-ATGTTTTCCTGTGCCCTGAG-3’ and 5’-TATGGTGGCAGAGTCAGTGG-3’.

### Statistical analyses

All the experimental data were analyzed by GraphPad Prism 8.0 (GraphPad Software Inc., San Diego, CA, United States). Results were expressed as mean values ± SD from at least 3 independent experiments. Statistics analysis was assessed using the two-way ANOVA. *p < 0.05 was considered as being significant (*p < 0.05, **p < 0.01, ***p < 0.001, ****p < 0.0001).

### Supplementary information

Supplemental figure legend

Figure S1

Figure S2

Figure S3

Figure S4

Figure S5

Figure S6

Figure S7

Figure S8

Figure S9

Figure S10

Original Data File

aj-checklist

## Data Availability

All datasets generated and analyzed during this study are included in this published article and its Supplementary Information files. A BioProject accession number (PRJNA953330) has been assigned to the sequencing data of this manuscript. Additional data are available from the corresponding author on reasonable request.
