# Supplementary material for: Targeting the E2F1/Rb/HDAC1 axis with the small molecule HR488B effectively inhibits colorectal cancer growth
Source: Cell Death Dis. 2023 Dec 7;14(12):801. doi: 10.1038/s41419-023-06205-0 (PMC10703885; doi:10.1038/s41419-023-06205-0)
Supplement: Supplementary file 1 — Supplemental figure legend [file 41419_2023_6205_MOESM1_ESM.docx]

**Fig. S1 The synthesis scheme of HR488B. a** Intermediate compound **2** was constructed from p-methoxyaniline and bromoacetonitrile, which was coupled with heptanedioic anhydride to yield acid **4** Then corresponding ester **5** was prepared by esterification of those acids. Subsequently, it was reacted with ammonium sulfide and triethylamine by heating to produce intermediate (**6**), and then cyclized with 2,2'-dichloroacetophenone to form intermediate **(8)**. Finally, treatment of methyl ester with hydroxylamine to afford the target compound HR488B. **b** HPLC purity: 99%. **c** The high-resolution mass spectrum (HRMS) of HR488B. HRMS (ESI): calcd for [C_24_H_26_ClN_3_O_4_S+H] ^+^ 487.1411, found 488.1402. HRMS was gathered on a Bruker MicroTOF-Q II LCMS instrument operating in electrospray ionization (ESI).

**Fig. S2 Nuclear Magnetic Resonance (NMR) spectroscopy of HR488B. a** 1H NMR (600 MHz, DMSO-d6) δ 10.30 (br s, 1H), 8.64 (br s, 1H), 8.03 (s, 1H), 7.78 (dd, J = 7.8, 7.8 Hz, 1H), 7.54 (dd, J = 7.8, 7.8 Hz, 1H), 7.42 – 7.36 (m, 2H), 7.23 (d, J = 9.0 Hz, 2H), 6.97 (d, J = 9.0 Hz, 2H), 5.09 (s, 2H), 3.76 (s, 3H), 2.06 (t, J = 7.2 Hz, 2H), 1.88 (t, J = 7.2 Hz, 2H), 1.49 – 1.44 (m, 2H), 1.41 – 1.36 (m, 2H), 1.16 – 1.11 (m, 2H). **b** 13C NMR (150 MHz, DMSO-d6) δ 172.32, 168.97, 166.11, 158.61, 149.98, 134.72, 132.76, 131.39, 130.9, 130.34, 129.55, 129.22, 127.38, 119.78, 114.76, 55.34, 50.50, 33.10, 32.12, 28.15, 24.91, 24.57. NMR spectra were recorded on a Bruker 600 MHz instrument and obtained as DMSO-d6 solutions (reported in ppm).

**Fig. S3 HR488B exhibits anti-tumor activity. a-e** H1299, A549, MCF-7, and HepG2 cells were seeded in 96-well plates and treated with HR488B (0.01, 0.05, 0.1, 0.25, 0.5, 1, 2.5, 5, 10, and 20 μM) for 72 h. The IC_50_ value of HR488B on non-small cell lung cancer, breast cancer, and liver cancer. **f** 293T, HCT116, and HT29 cells were treated with various concentrations of HR488B (0, 0.1, 0.2, 0.5, 1 μM), and cell viability was measured at 72 h using CCK-8 assay.

**Fig. S4 The binding pattern of HR488B in the active site of HDAC2 (PDB 7ZZS). a** The 3D binding mode of HR488B in the active site of HDAC2. The protein and ligand HR488B and HDAC2 are shown by cartoon and stick respectively with key residues labeled and demonstrated as green sticks, and the hydrogen bonds are labeled by red dashed lines. Docking Score of HR488B and HDAC2 is shown. **b** Diagrammatic illustration of interaction between HDAC2 binding site residues and HR488B by BIOVIA Discovery Studio Visualizer software. Ligand is presented by gray line, green dashed line is conventional hydrogen bonds, light green dashed line is Van der Waals, and light pink dashed line is Pi-Alkyl.

**Fig. S5 Docking score of SAHA and HR488B and interaction mode of HR488B. a** Docking score of SAHA and HR488B. **b** Hydrogen bond analysis for each protein−ligand system. **c** Depicted the interaction between HDAC1 and HR488B at 3 ns, conventional hydrogen bonds (green) and carbon hydrogen bonds (light green) were marked in red. **d** 2D binding model at 5.5 ns. **e** 2D binding model at 8 ns.

**Fig. S6 HR488B alters gene expression in CRC. a** Venn diagram showing the overlap of 12451 genes from HR488B-treated group and DMSO control group. **b** Heatmap illustrating differentially expressed genes between DMSO and HR488B (10 μM) groups.

**Fig. S7 Overexpression of HDAC1 and E2F1 antagonize the anti-cancer effects of HR488B.** CCK-8 analyzing the effect of HR488B (0.5μM) for 24h on cell viability after overexpression of HDAC1 and E2F1 protein in HCT116 cells. All data are shown as mean ± SD, n=3, two-way ANOVA, **p* < 0.05, ** *p* < 0.01, *** *p* < 0.001.

**Fig. S8 E2F1 is essential for HR488B-induced ROS accumulation. a** HCT116 cells were transfected with siRNAs targeting siE2F1 or siNC, 24 h later, cells were treated with DMSO or HR488B (0.5 μM) for 24 h, Intracellular ROS was measured by flow cytometry after 10 μM DCFH-DA staining. **b** Statistical analysis of the percentage of ROS generation. All data are shown as mean ± SD, n=3, two-way ANOVA, **p* < 0.05, ** *p* < 0.01.

**Fig. S****9 The effect of HR488B on the transcriptional expression and protein stability of E2F1. a** HCT116 cells were treated with DMSO or HR488B (0.2, 0.5, and 1μM) for 24 h, and mRNA expression levels of E2F1 were determined by Real-time PCR assays. **b** HCT116 cells were treated DMSO or 1μM HR488B for 24 h, followed by the addition of the protein synthesis inhibitor cycloheximide (CHX; 50 mg/mL). Stability of endogenous E2F1 was examined by Western blot assay. **c** The statistical result of (**b**)**.** All data are shown as mean ± SD, n=3, two-way ANOVA, **p* < 0.05, ** *p* < 0.01, *** *p* < 0.001.

**Fig. S10 HR488B remarkably decreases the phosphorylation of Rb protein by inhibiting the expression of CDK4 in HCT116 cells. a** HCT116 cells were treated with DMSO or indicated concentrations of HR488B (0.2, 0.5, and 1 μM) for 24 h, respectively, and the expression of CDK4, Cyclin D1, p-Rb, and Rb was detected by Western blot analysis, and β-actin was detected as the endogenous loading control, accordingly. **b** The statistical result of (**a**).**c** HCT116 cells were transfected with siRNAs targeting siCDK4 or siNC, and 48 h later, cells were treated with DMSO or HR488B (0.5 μM) for 24 h, and then the expression of CDK4, p-Rb, and Rb protein was determined by Western blot analysis, and β-actin was detected as the endogenous loading control, accordingly. **d** The statistical result of (**c**). All data are shown as mean ± SD, n=3, two-way ANOVA, **p* < 0.05, ** *p* < 0.01, *** *p* < 0.001.
