# Supplementary figures and images for: Targeting the E2F1/Rb/HDAC1 axis with the small molecule HR488B effectively inhibits colorectal cancer growth
Source: Cell Death Dis. 2023 Dec 7;14(12):801. doi: 10.1038/s41419-023-06205-0 (PMC10703885; doi:10.1038/s41419-023-06205-0)

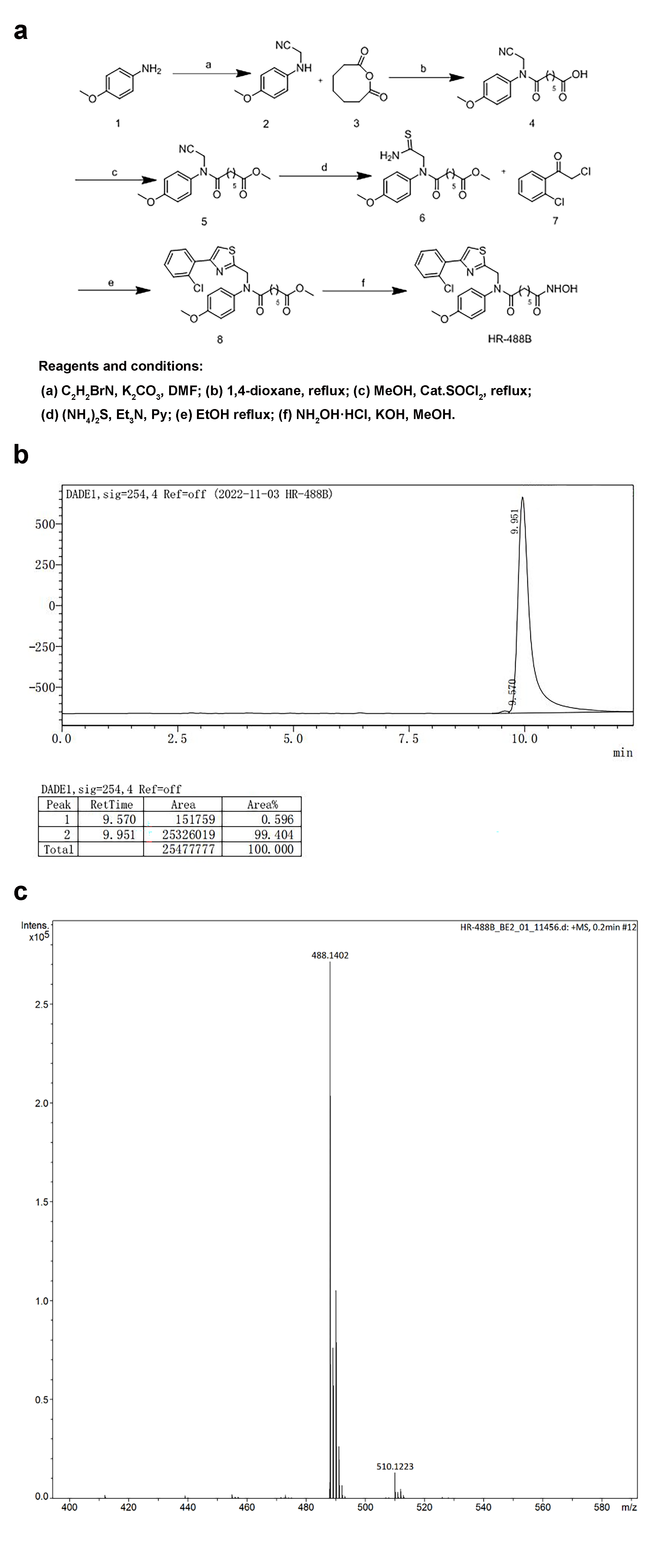

Supplement: Supplementary file 2 — Figure S1 [file 41419_2023_6205_MOESM2_ESM.tif]

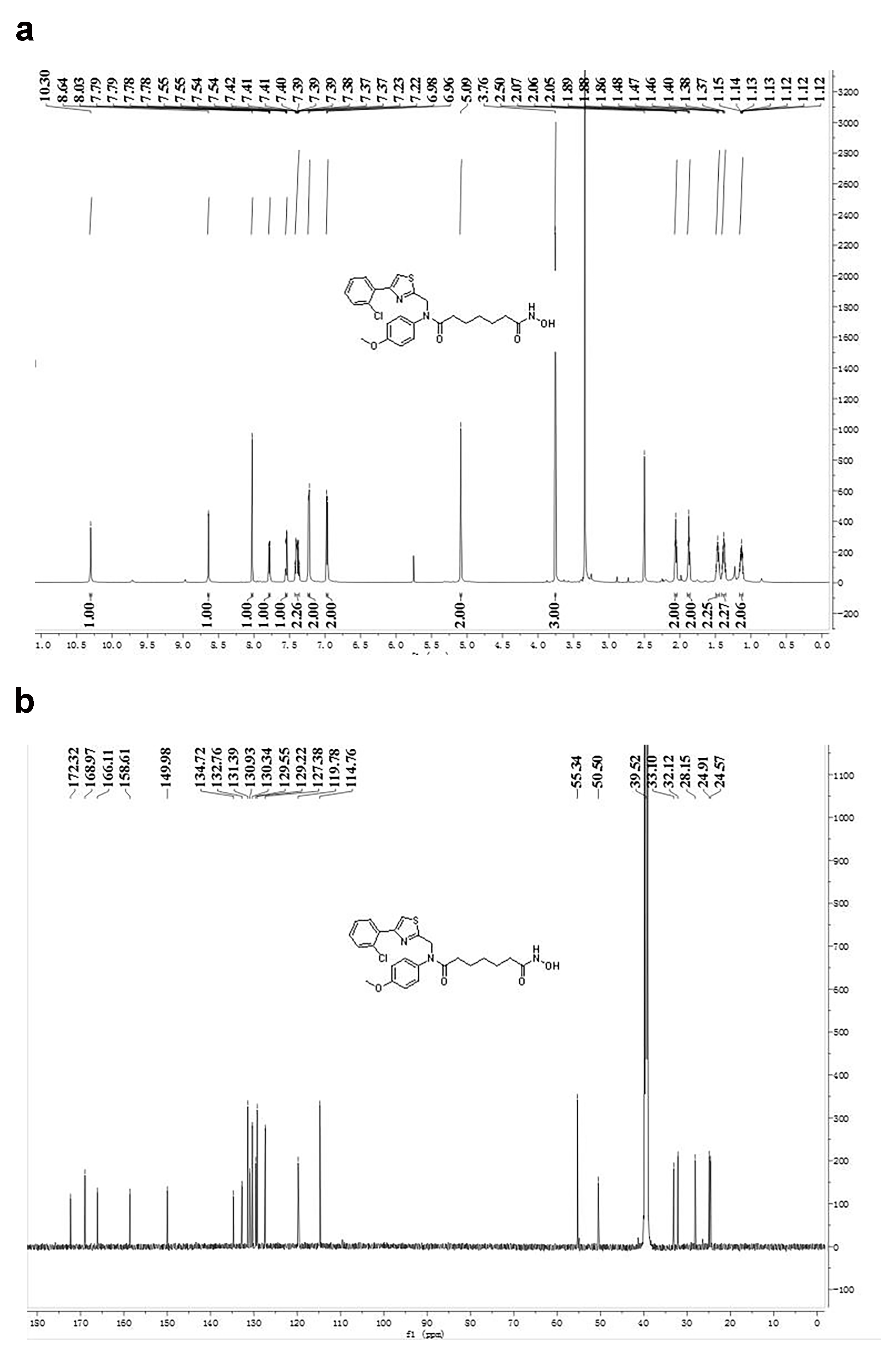

Supplement: Supplementary file 3 — Figure S2 [file 41419_2023_6205_MOESM3_ESM.tif]

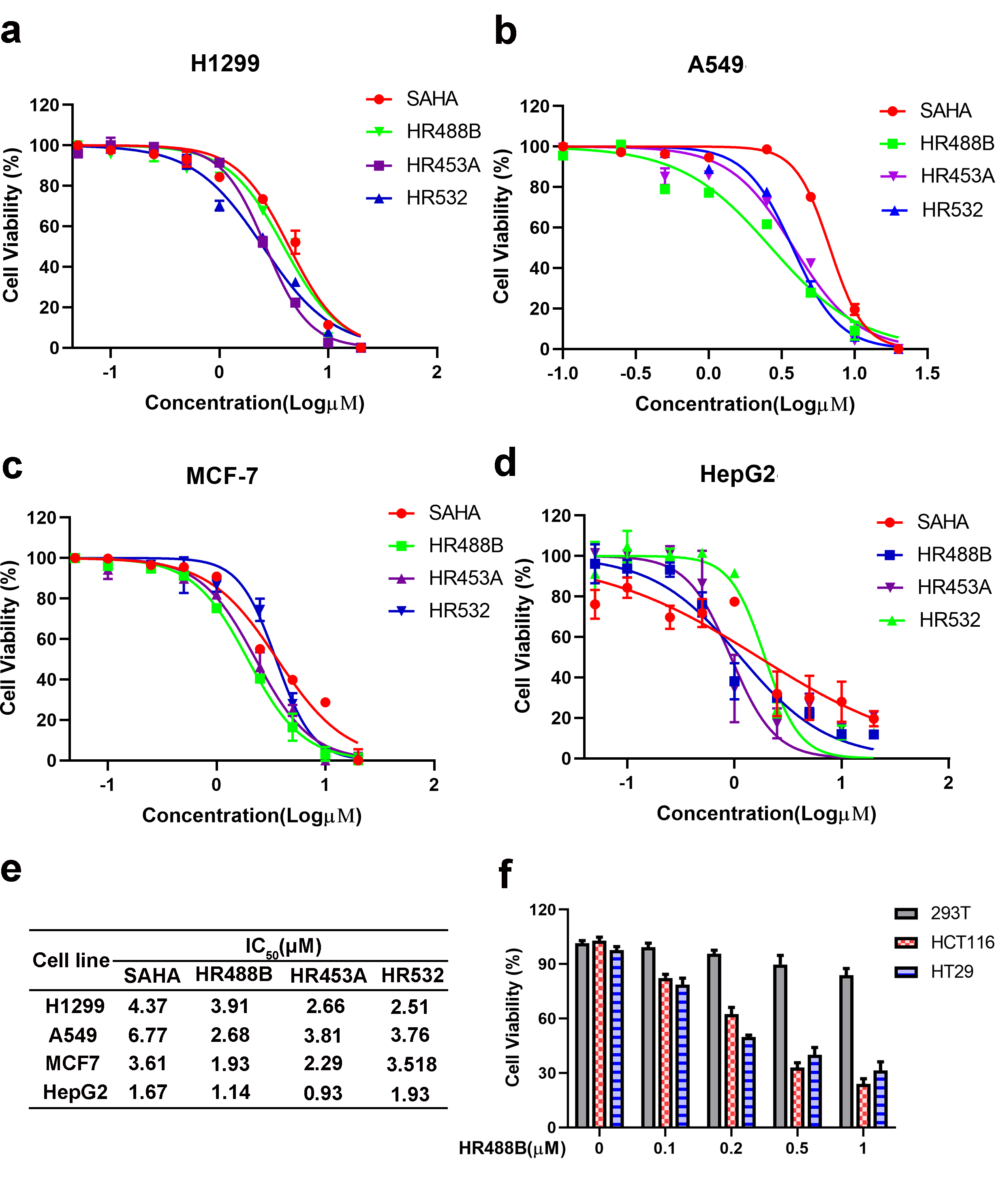

Supplement: Supplementary file 4 — Figure S3 [file 41419_2023_6205_MOESM4_ESM.tif]

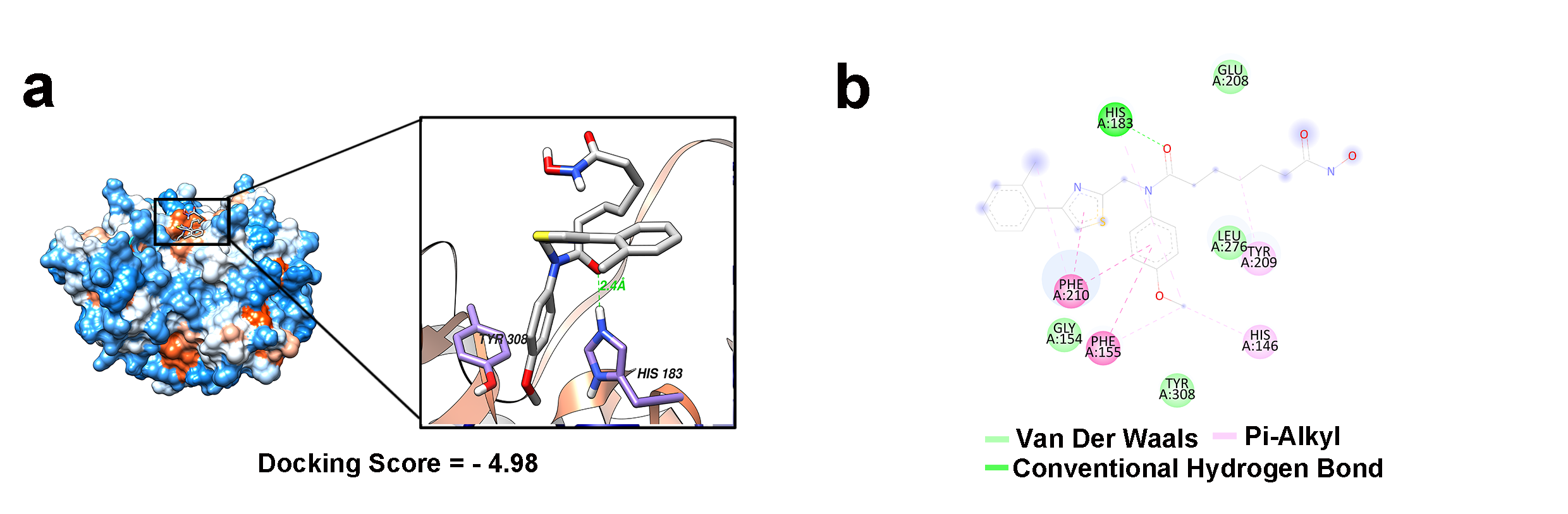

Supplement: Supplementary file 5 — Figure S4 [file 41419_2023_6205_MOESM5_ESM.tif]

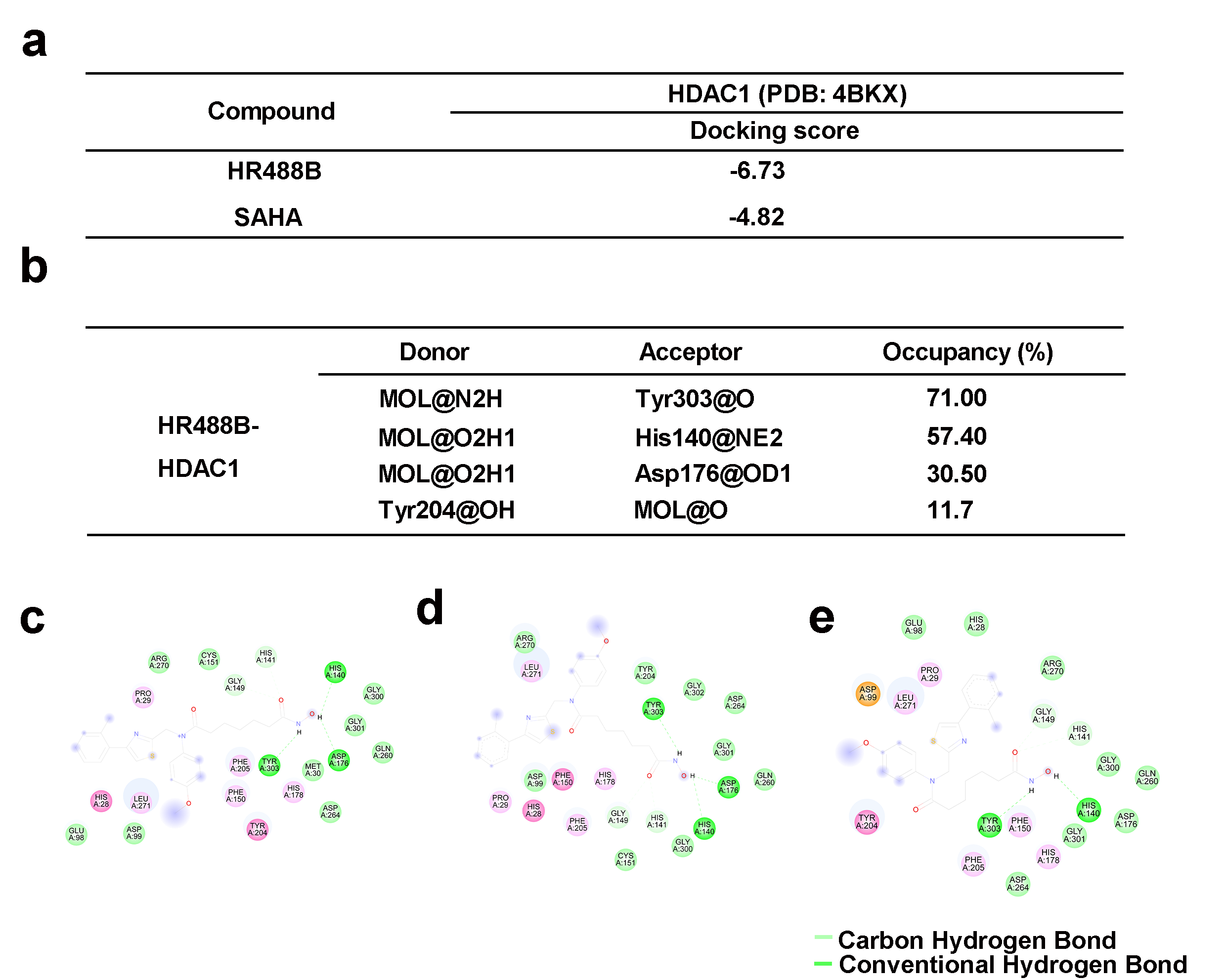

Supplement: Supplementary file 6 — Figure S5 [file 41419_2023_6205_MOESM6_ESM.tif]

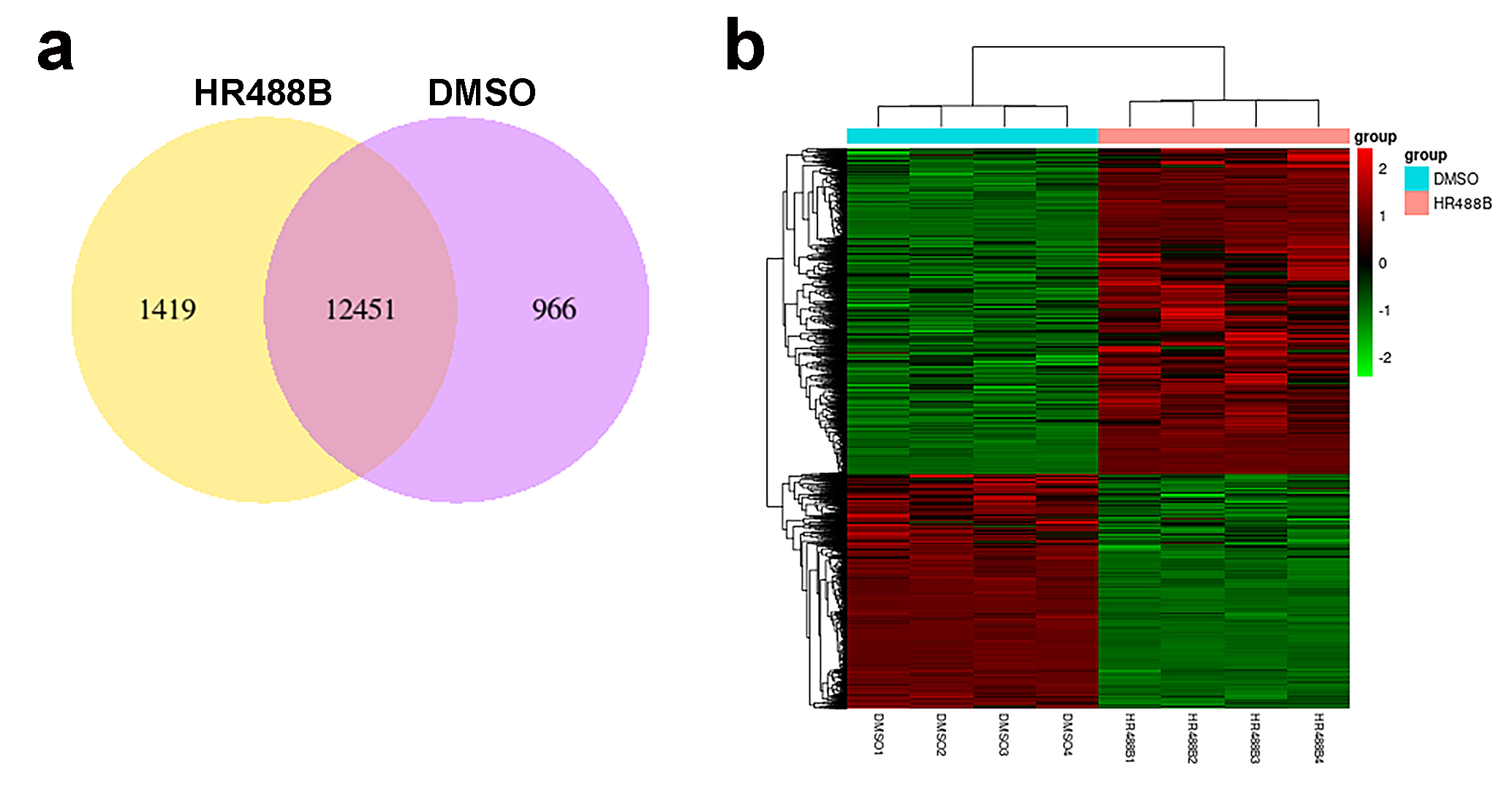

Supplement: Supplementary file 7 — Figure S6 [file 41419_2023_6205_MOESM7_ESM.tif]

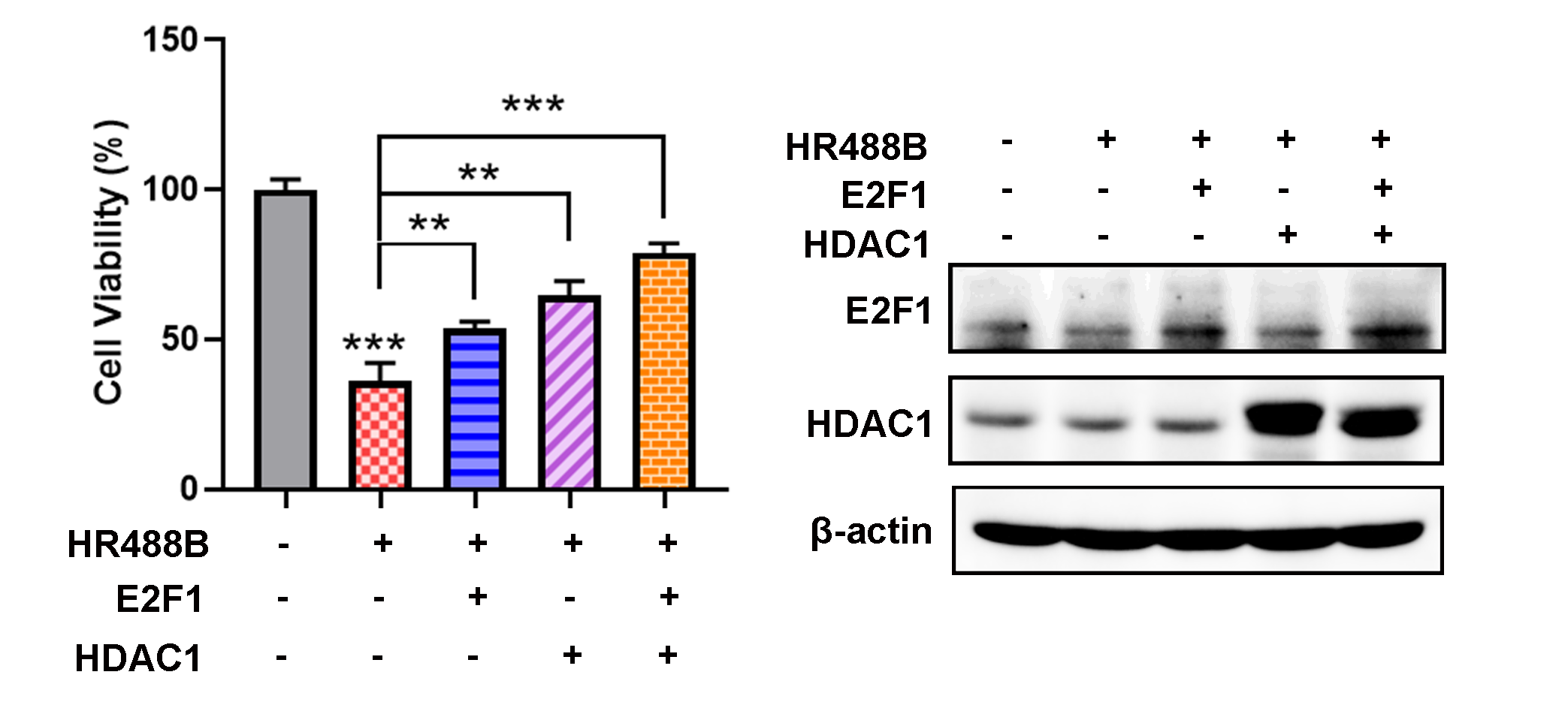

Supplement: Supplementary file 8 — Figure S7 [file 41419_2023_6205_MOESM8_ESM.tif]

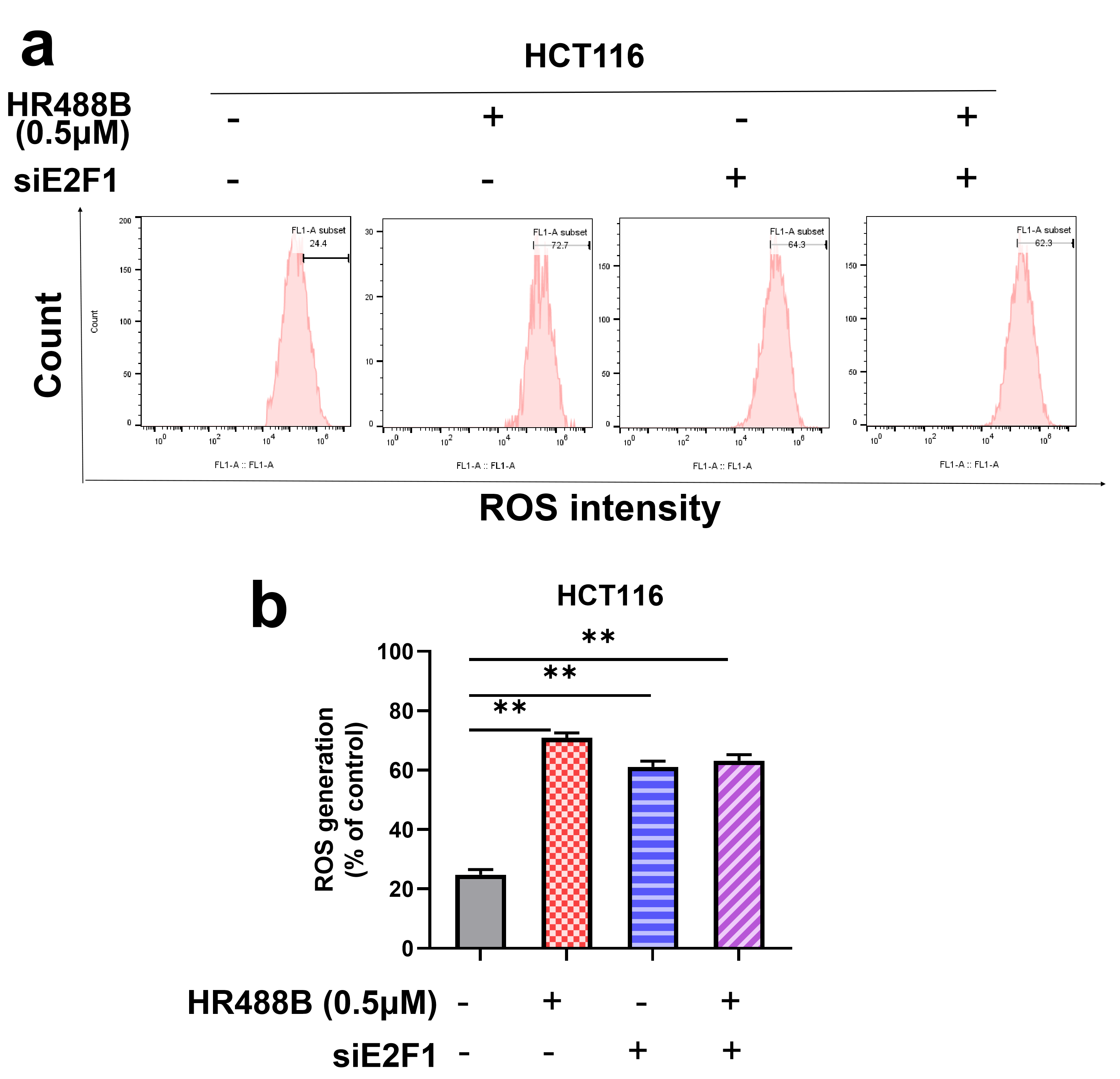

Supplement: Supplementary file 9 — Figure S8 [file 41419_2023_6205_MOESM9_ESM.tif]

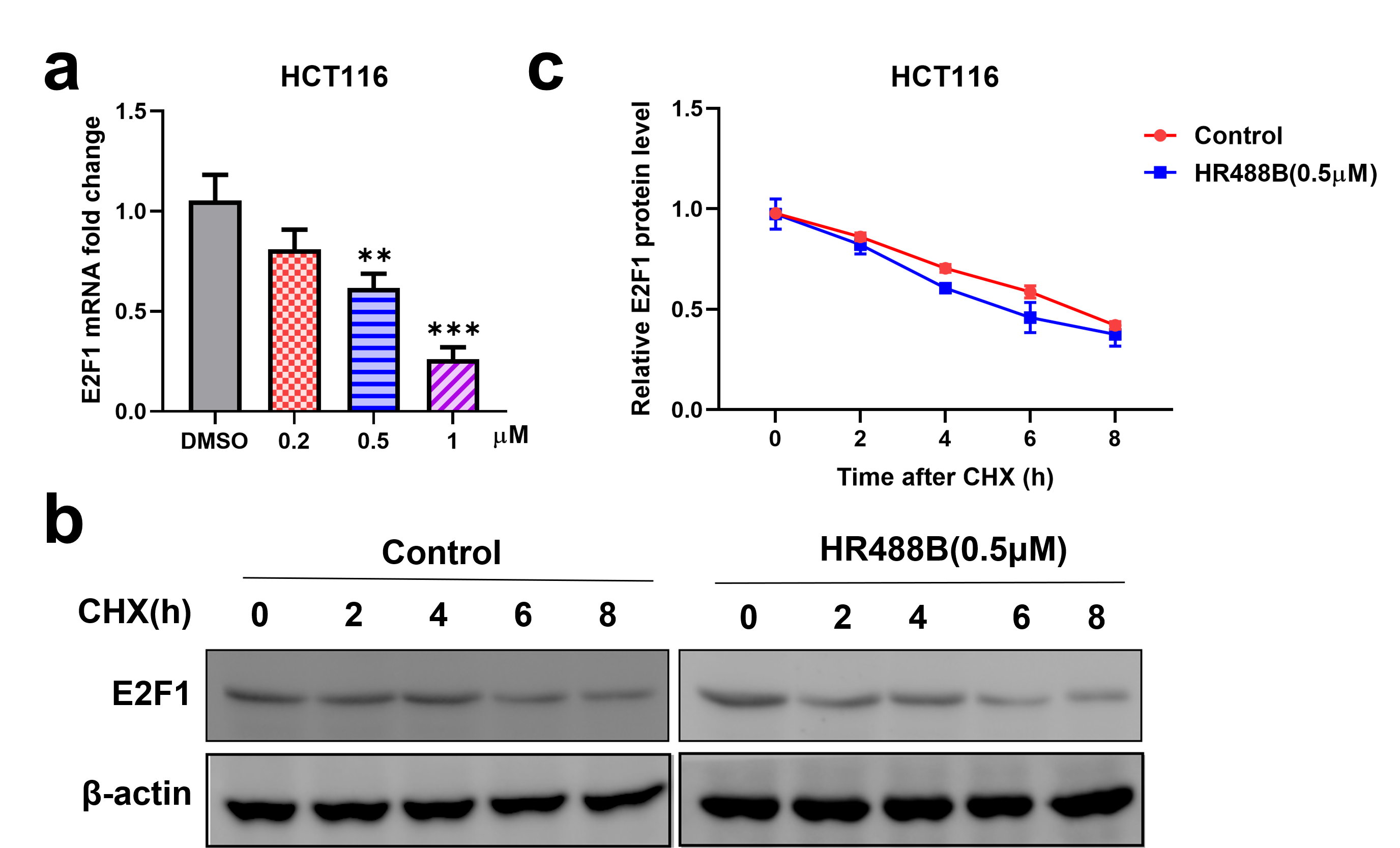

Supplement: Supplementary file 10 — Figure S9 [file 41419_2023_6205_MOESM10_ESM.tif]

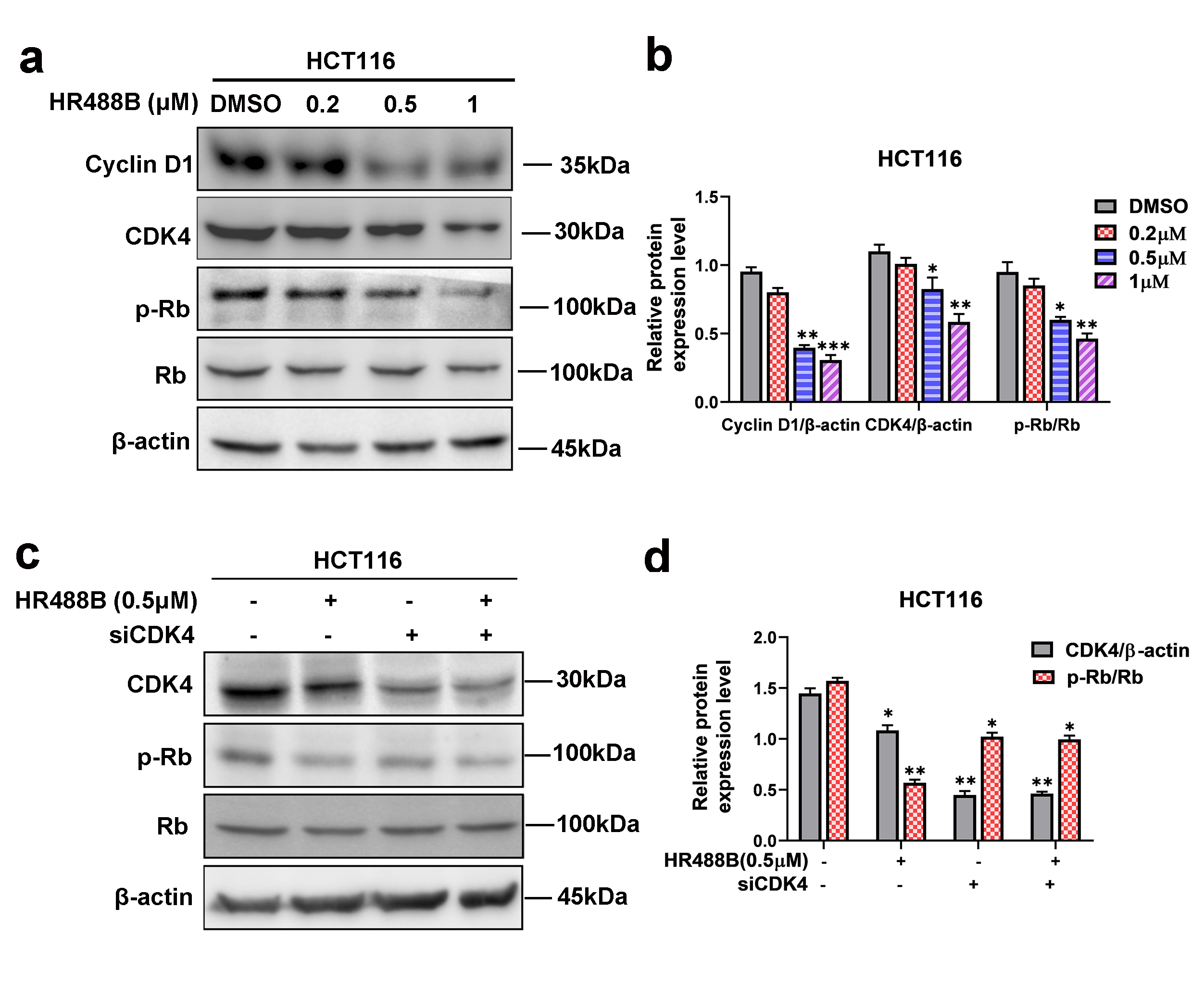

Supplement: Supplementary file 11 — Figure S10 [file 41419_2023_6205_MOESM11_ESM.tif]

**Fig1d**

**
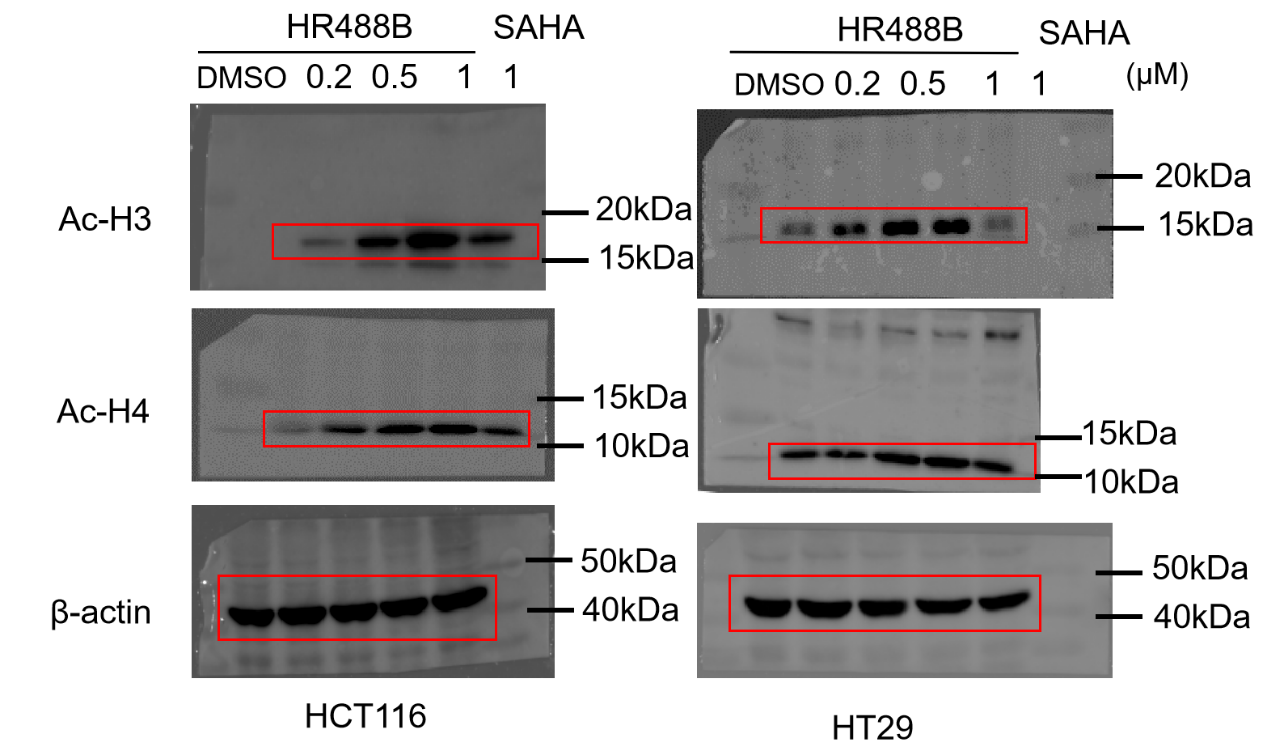
**

**Fig2h**

**
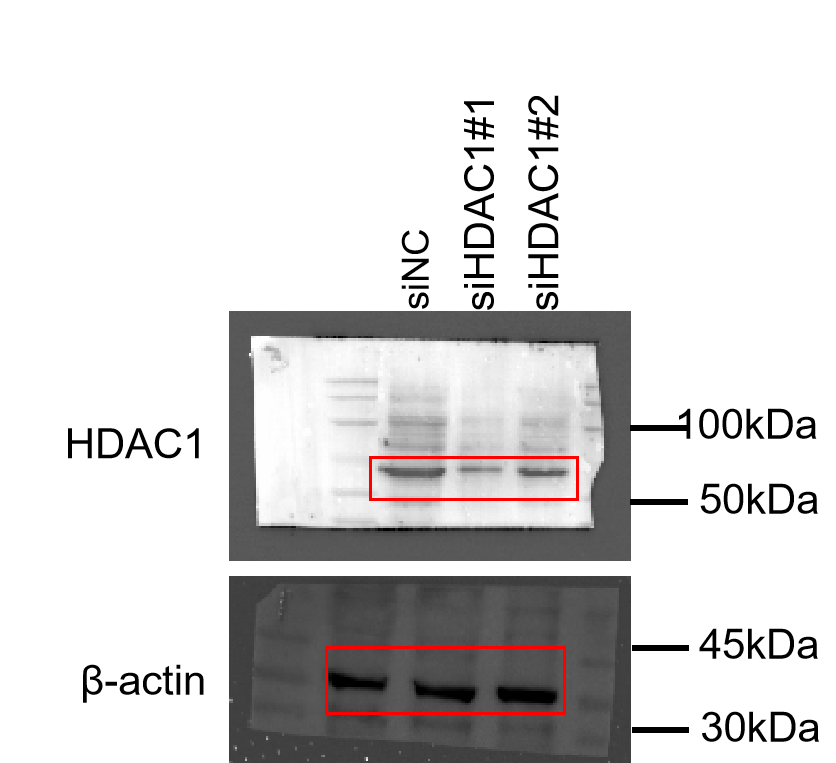
**

**Fig2i**

**
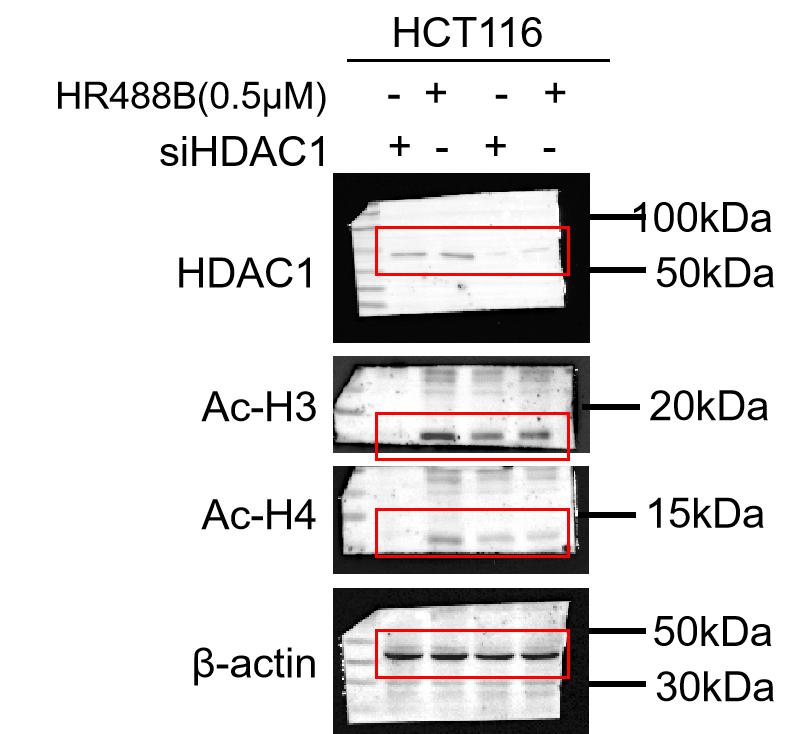
**

**Fig3g**

**
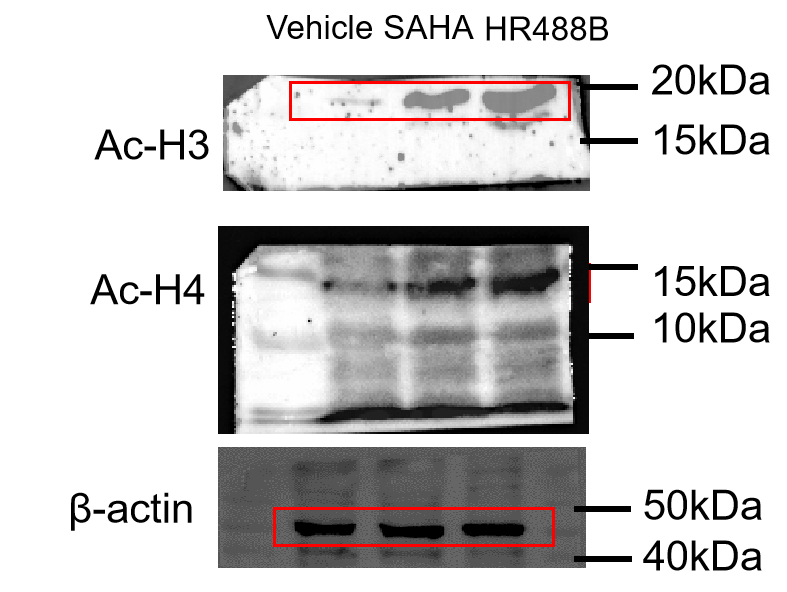
**

**Fig4g**

**
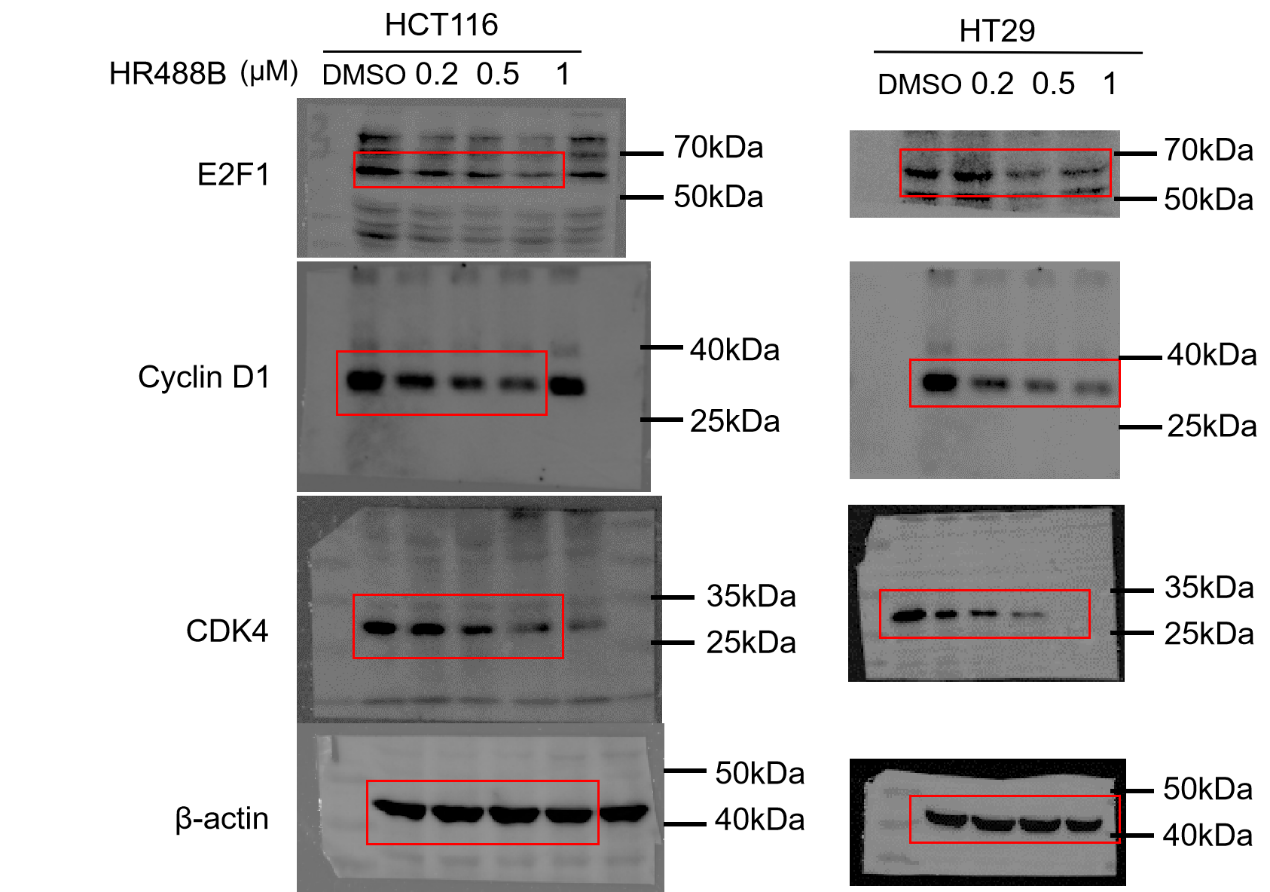
**

**Fig5c**

**
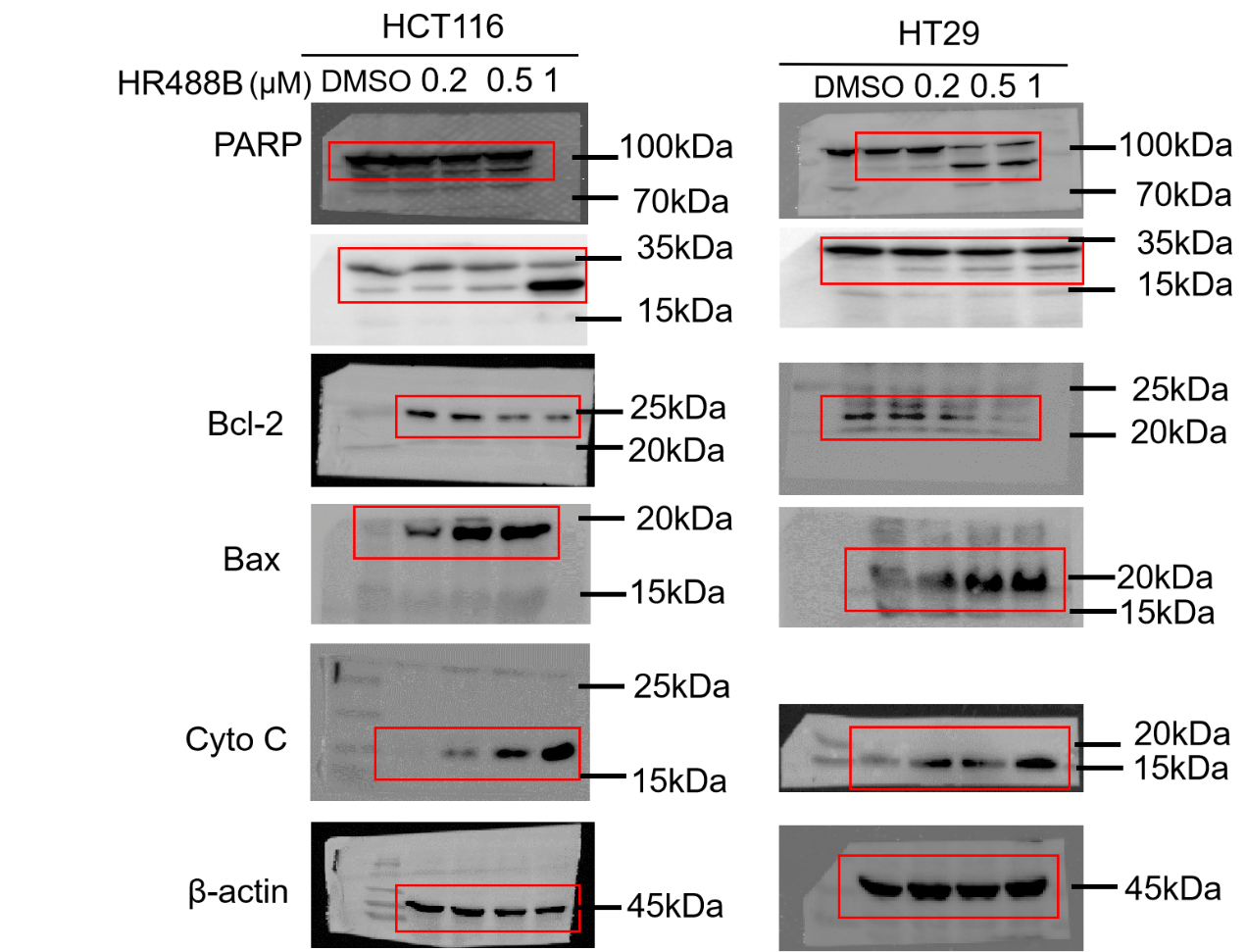
**

**Fig5l**

**
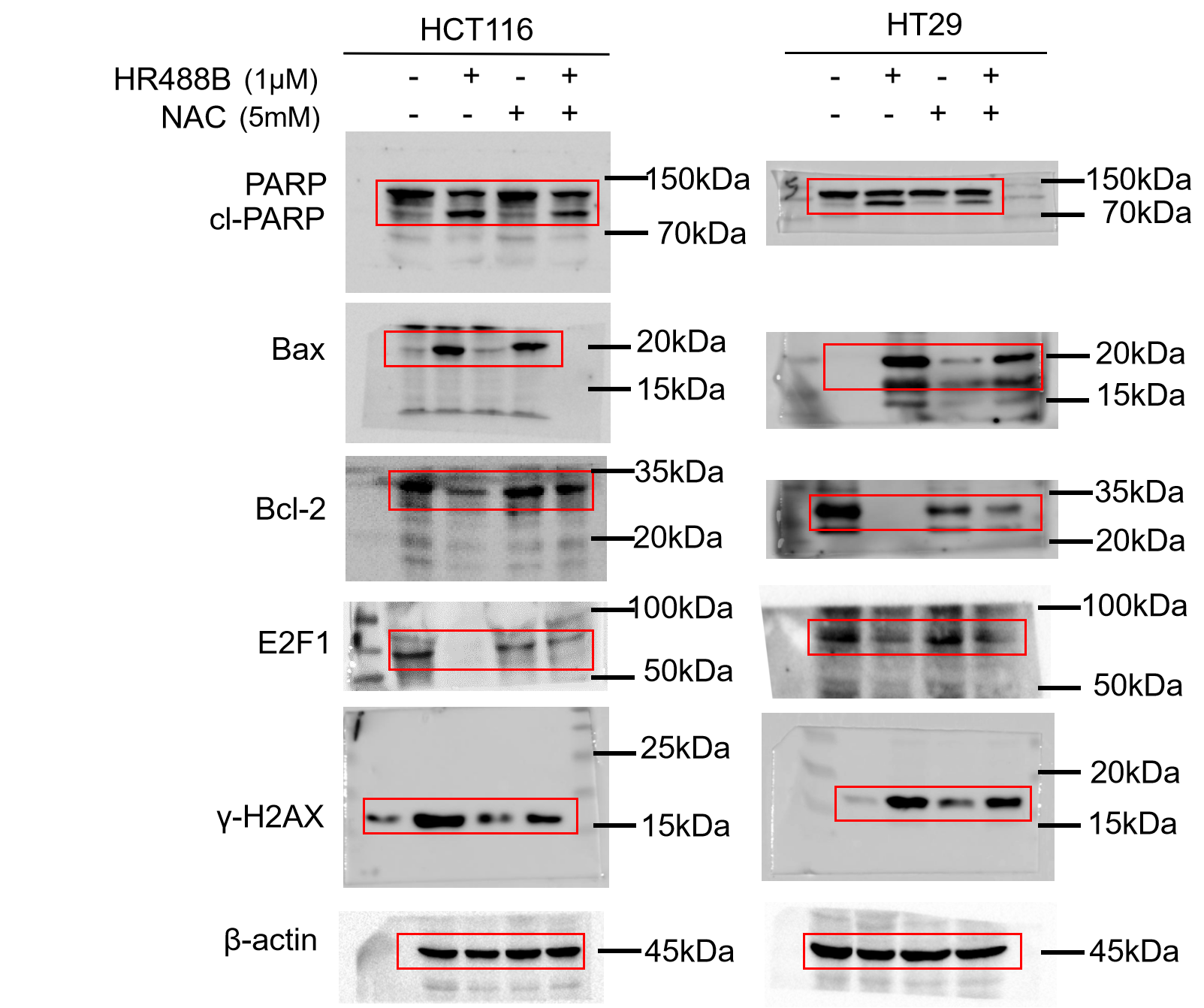
**

**Fig6c**

**
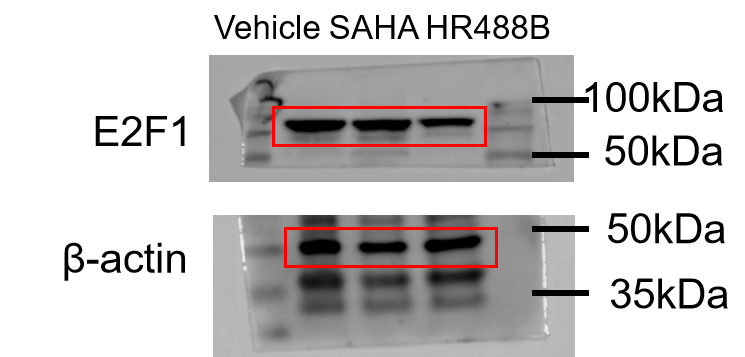
**

**Fig6e**


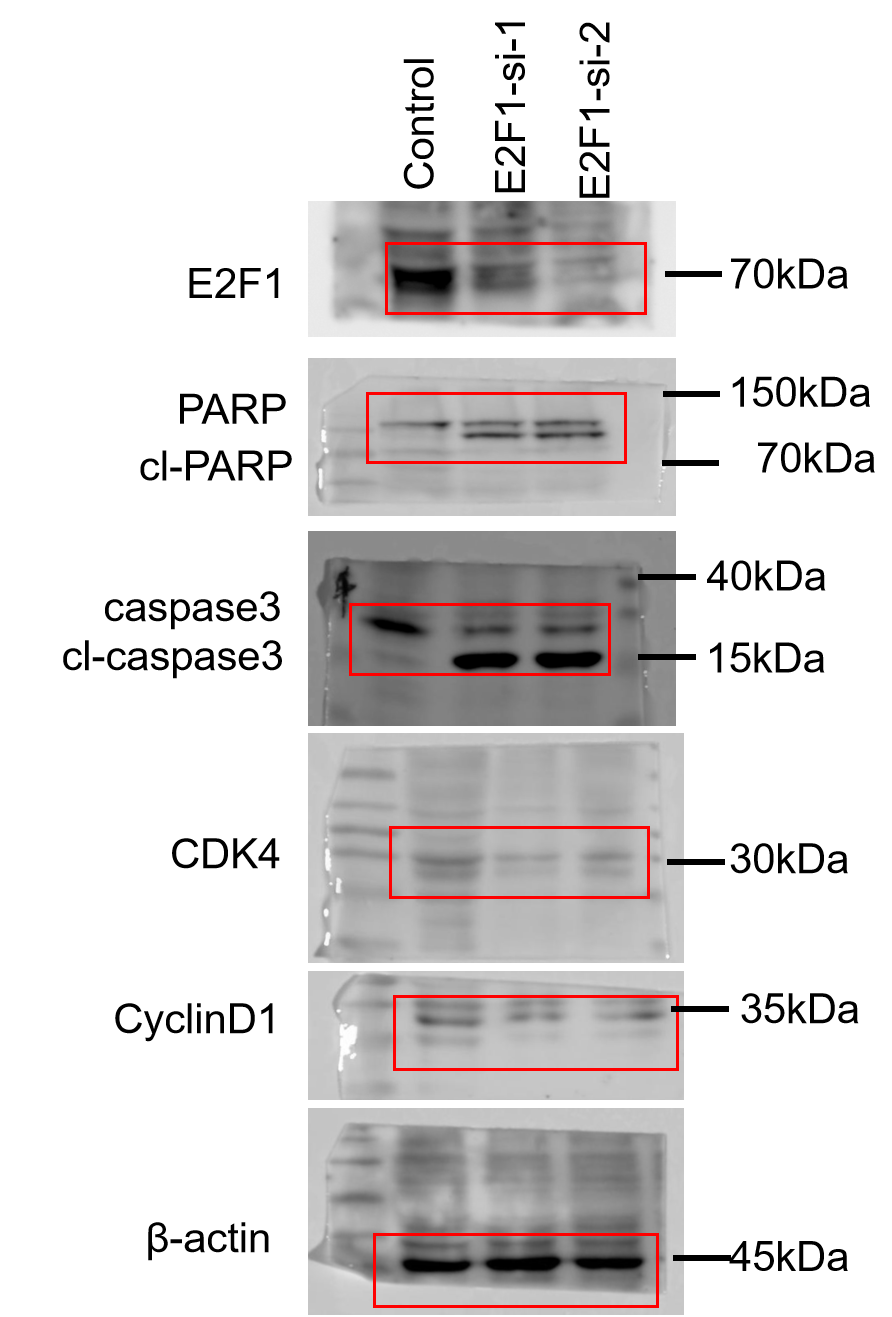


**Fig6m**

**
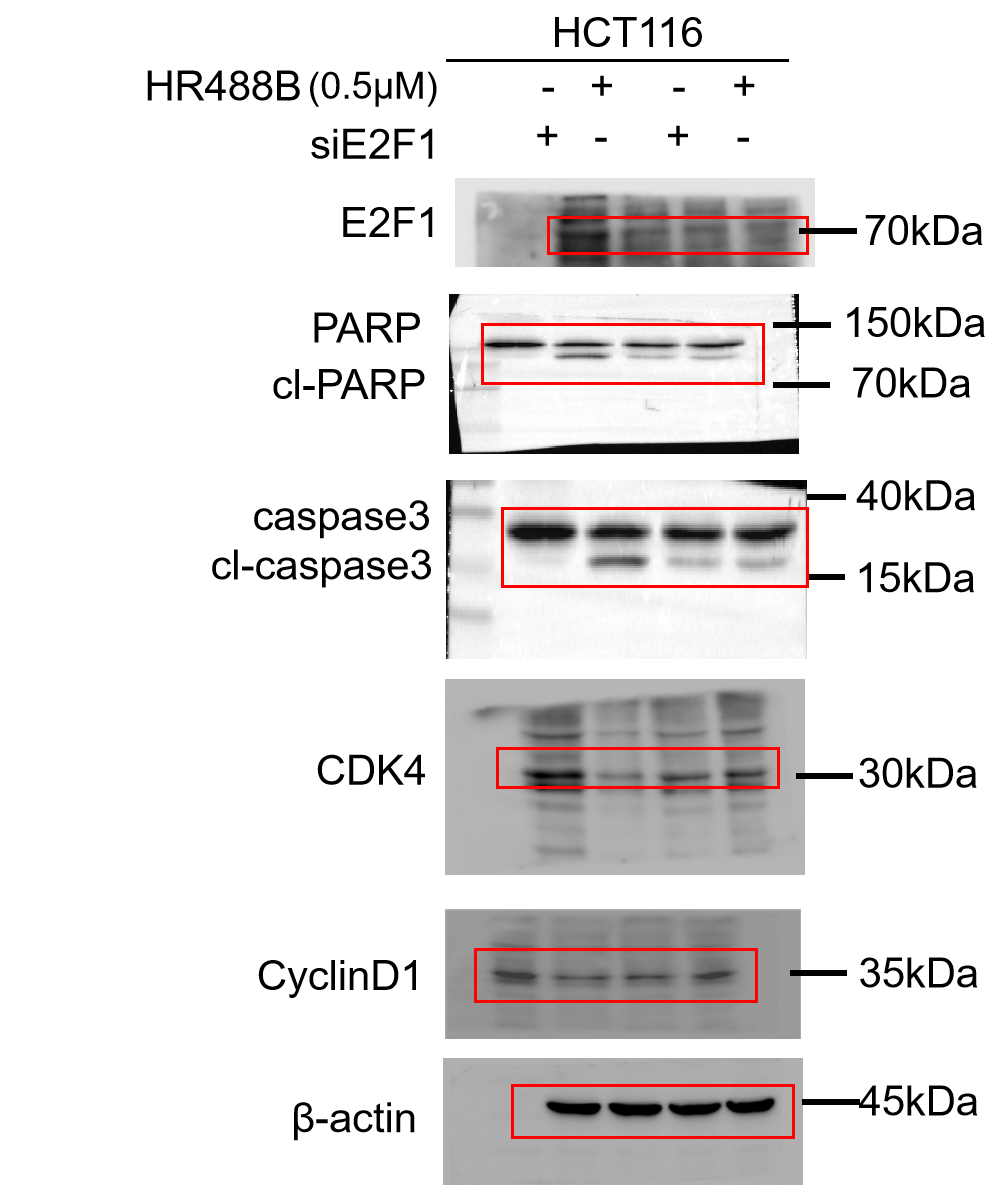
**

**Fig6o**

**
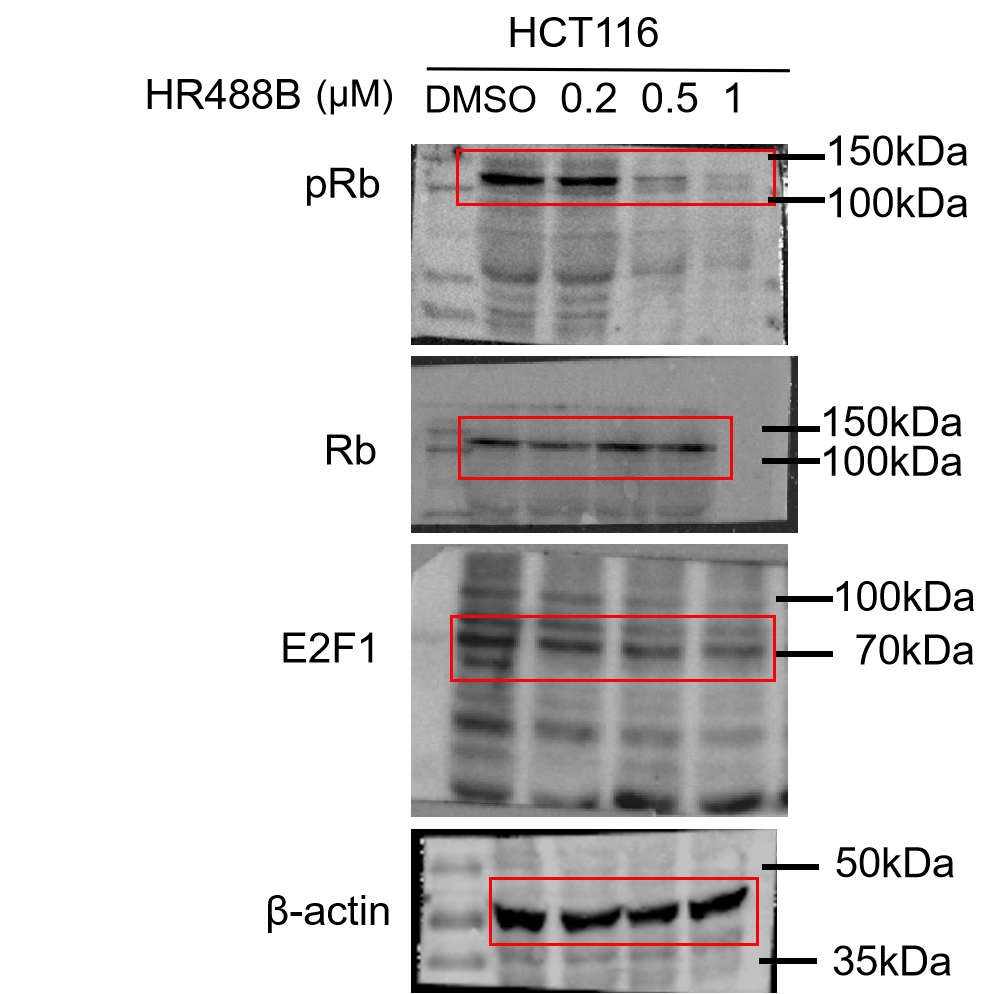
**

**Fig6p**

**
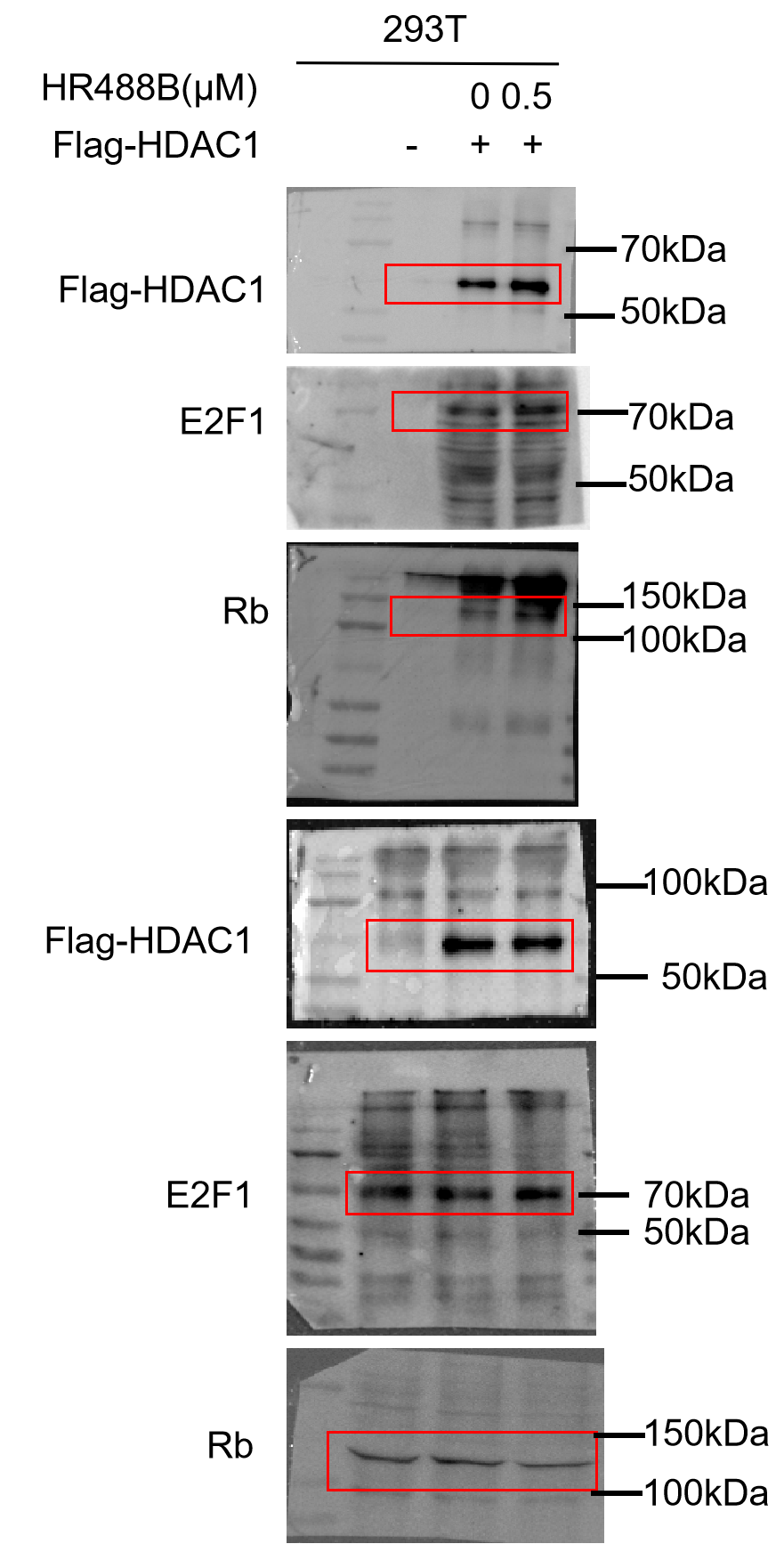
**

**FigS7**

**
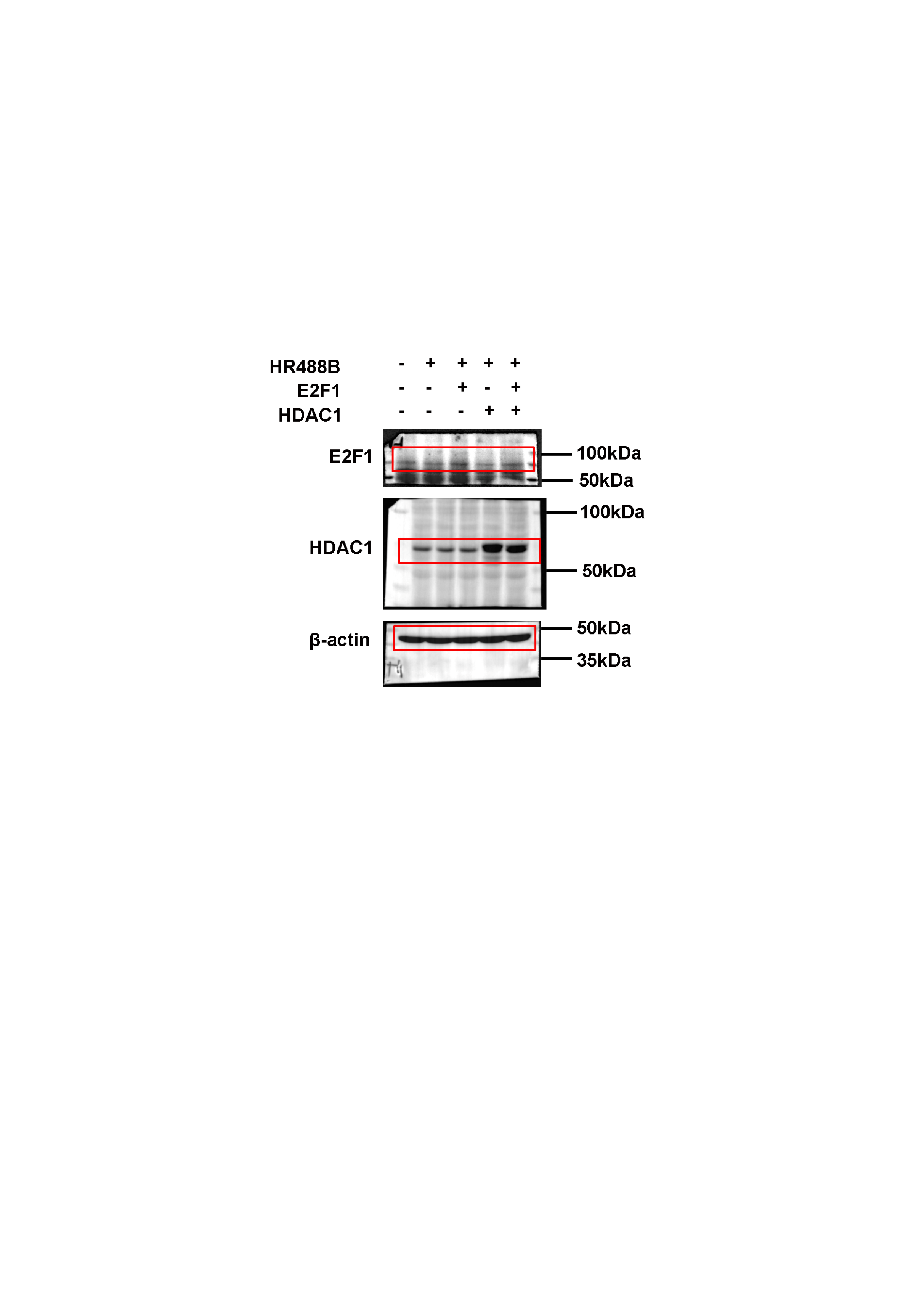
HCT116**

**FigS9b**

**
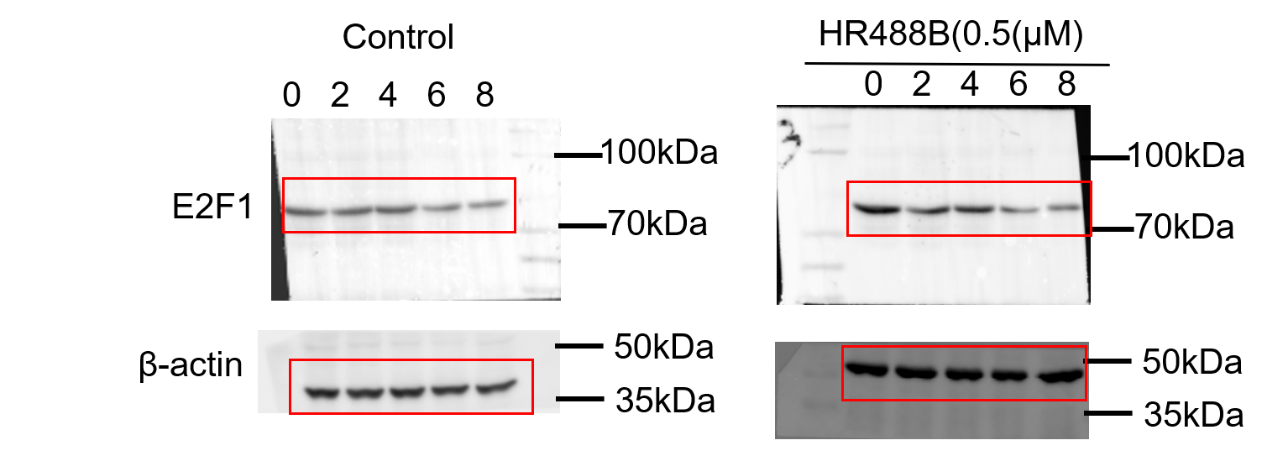
**

**FigS10a**

**
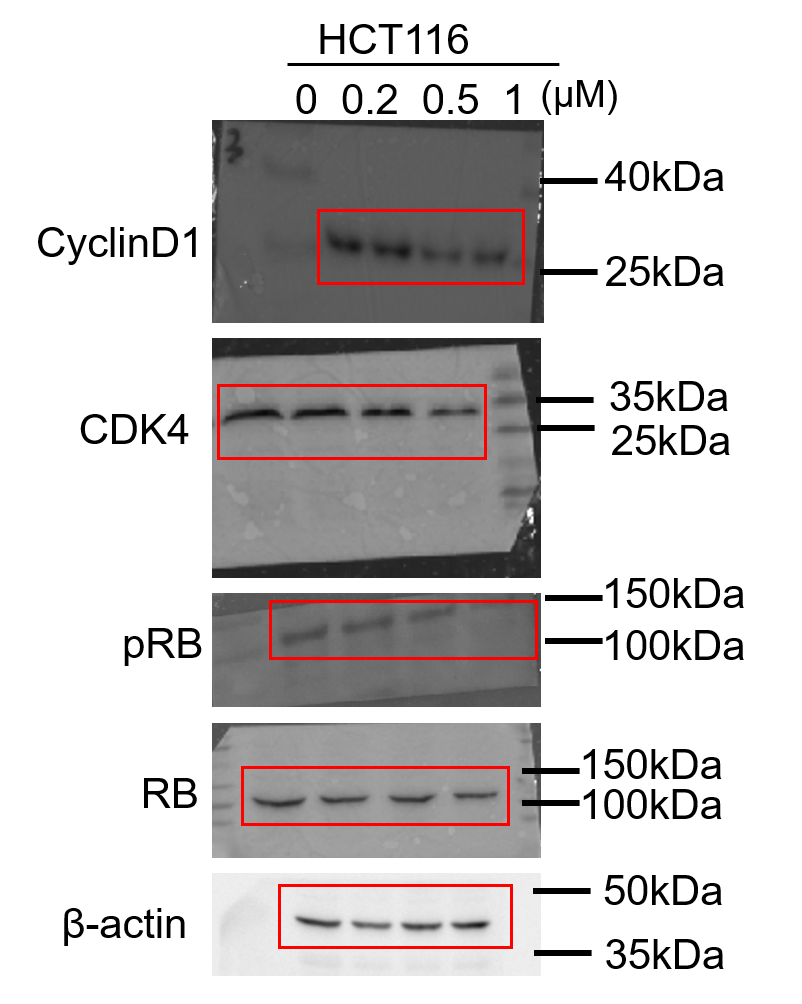
**

**FigS10c**

**
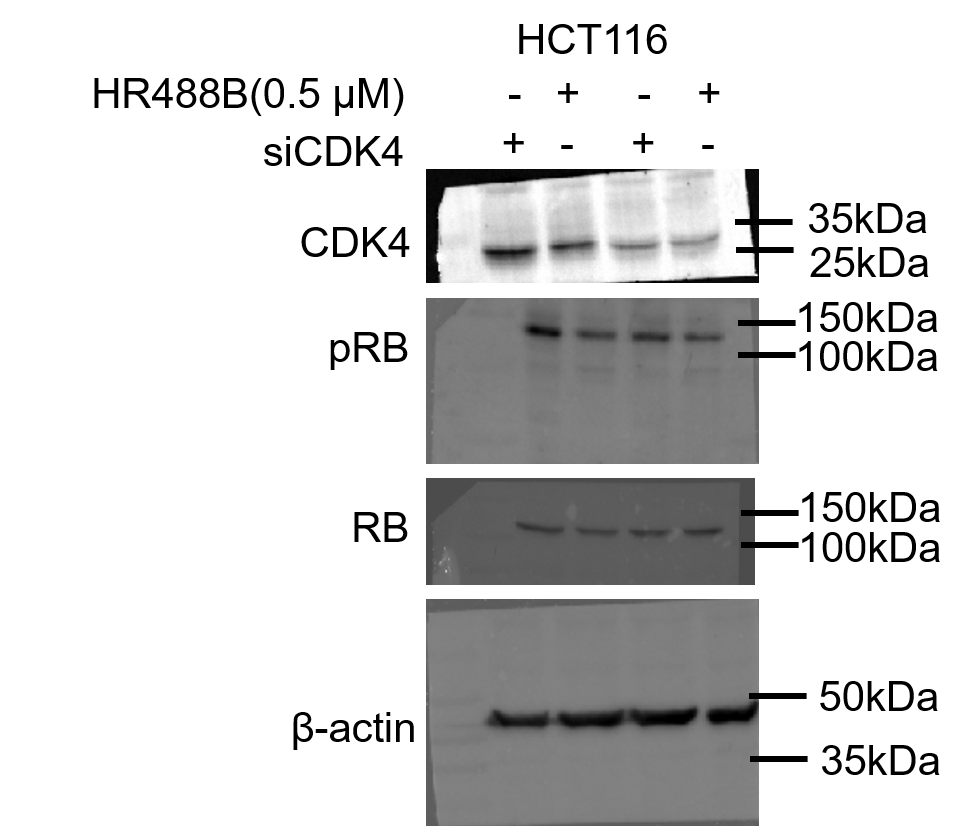
**

Supplement: Supplementary file 12 — Original Data File [file 41419_2023_6205_MOESM12_ESM.docx]
